# Identification and exploration of anticancer activity of novel peptides isolated from the edible bivalve *Callista chione* in hepatic and colon cancer cell lines

**DOI:** 10.1016/j.toxrep.2025.101915

**Published:** 2025-01-27

**Authors:** Ahmed A.A. Hussein, Hend Okasha, Mohamed ElZallat, Samah I. Ghoname, Mohamed R. Habib, Olfat A. Hammam, Ehab El-Dabaa, Maha B. Salem

**Affiliations:** a Departments of Medical Malacology, Theodor Bilharz Research Institute, Warrak El-Hadar, Imbaba, Giza 12411, Egypt; b Biochemistry and Molecular Biology Department, Theodor Bilharz Research Institute, Warrak El-Hadar, Imbaba, Giza 12411, Egypt; c Immunology Department, Theodor Bilharz Research Institute, Warrak El-Hadar, Imbaba, Giza 12411, Egypt; d Pathology Department, Theodor Bilharz Research Institute, Warrak El-Hadar, Imbaba, Giza 12411, Egypt; e Pharmacology Department, Theodor Bilharz Research Institute, Warrak El-Hadar, Imbaba, Giza 12411, Egypt

**Keywords:** Marine sources, Anticancer, Apoptosis, Autophagy, Bioactive peptides, FGF19-FGFR4 axis, FPLC

## Abstract

**Background:**

The requirement for relevant and safe drugs for cancer treatment is considered a challenge. Recently, marine isolated compounds with various therapeutic targets have attracted many researchers.

**Aim:**

Isolation and identification of potential anticancer peptides from edible *Callista chione* soft tissues.

**Methodology:**

*C. chione* specimens were collected and peptides were extracted, purified with FPLC, and tested on normal (hepatocytes and VERO) and cancer (HepG2, and HT-29) cells. Bioactive fractions were tested by tandem mass spectrometry.

**Results:**

Five different fractions were purified according to ionic charges and two fractions (4 and 5) showed a potent anticancer activity with a total anticancer score threshold of ≥ 0.5, and hydrophilicity mean of 1.75 that related to stability and solubility. The apoptotic and autophagy-related markers were significantly up-regulated in both HepG2 and HT-29 cells treated with IC_50_ of bioactive peptides’ fractions 4 and 5, explaining their underlying mechanism of action.

**Conclusion:**

Natural source peptides derived from the soft tissue of *C. chione* could be exploited for the treatment of cancers and a deep in silico study will be performed for further investigation and deep function identification.

## Highlights

• Anticancer peptides isolated from *Callista chione* soft tissues using extraction and purification techniques.
• Bioactive fractions identified by tandem mass spectrometry, revealing peptides with potential anticancer activity.
• Peptides inhibited the FGF19–FGFR4 pathway in HepG2 and HT-29 cells, reducing proliferation and tumor growth.
• Peptides showed a favorable safety profile, selectively targeting cancer cells while sparing normal cells from toxicity.

## Graphical Abstract

**Figure:**
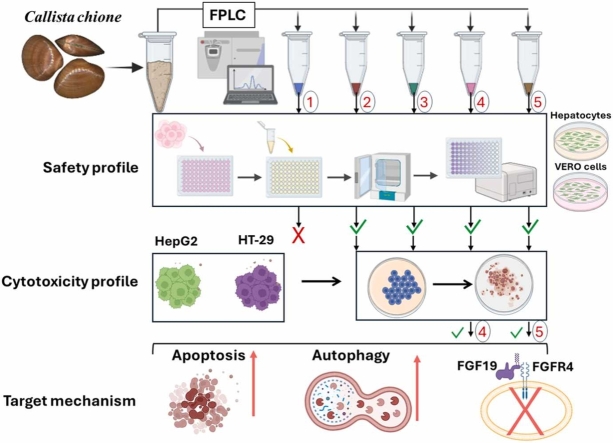

## 1 Introduction

Liver and colon cancers rank among the most prevalent types of cancer affecting the digestive system worldwide [2]. Hepatocellular carcinoma (HCC) is amongst the most frequent cancers and it ranks the second leading cause of cancer mortality worldwide [11] with 700,000 deaths and about 800,000 new cases annually [4]. Multiple factors contribute to the development of HCC, including chronic hepatitis B virus infection, heavy alcohol consumption, obesity, diabetes, and smoking[32]. Colorectal cancer (CRC) is another prevalent cancer, ranking the third most prevalent kind of cancer worldwide [3]. Risk factors for CRCs include smoking, obesity, a sedentary lifestyle, and a diet deficient in fruits and vegetables[27].

Considering the significant prevalence and severity of HCC and CRC, elucidating the mechanisms underlying tumor suppression is critical. Autophagy and apoptosis work together as essential mechanisms in suppressing tumors. Autophagy helps in breaking down harmful oncogenic substances, thus inhibiting cancer formation, whereas apoptosis prevents the survival of malignant cells [33]. Given the multifaceted roles of both autophagy and apoptosis in maintaining cellular homeostasis, it's not unexpected to find a significant interplay between them, facilitating their coordinated regulation [17]. Among these crosstalks, the beclin 1-Bcl-2 interaction [21]; and caspase-mediated beclin 1 cleavage are considered vital tumor suppressor pathways [36]. In addition, the fibroblast growth factors ligands and receptors (FGF and FGFR) play critical roles in tumorigenesis, which is found in different multicellular organisms [18].

Fibroblast growth factors (FGFs) transmit signals through four transmembrane tyrosine kinase receptors, referred to as fibroblast growth factor receptors (FGFRs), which include FGFR1, FGFR2, FGFR3, and FGFR4 [9]. FGFR4 exhibits significant activation in certain cancer types, often linked to its specific activator, FGF19. The FGF19-FGFR4 signaling axis impacts various cellular processes, such as proliferation, migration, metabolism, and differentiation [19].

Multiple treatment possibilities for managing HCC and CRC such as curative resection are available. However, these therapeutic possibilities come with numerous adverse effects that can be severe to patients, such as severe pain and anemia, in addition to their considerable financial burden [16], [39]. In addition to the surgical possibility, chemotherapy remains one of the most frequently employed treatment modalities regardless of undesirable side effects such as drug resistance, inadequately targeted distribution, and normal cell death. These side impacts have diminished these medications' effectiveness owing to therapeutic failure [28].

Recently there has been an increasing demand for safer, more targeted cancer treatments. In this respect, peptides have emerged as promising candidates in the pharmaceutical industry. Notably, peptides offer an alternative therapeutic approach by mimicking natural metabolic processes in the body, thus exhibiting lower toxicity and better selectivity [35]. The rich biodiversity of the marine ecosystem provides a vast array of species that serve as valuable sources for discovering novel functional proteins and peptides, which hold the potential for treating various diseases, including cancer (Q.-T. [40]). Globally, around 49 active substances derived from marine sources, or their derivatives, have either been approved for market use or are currently in clinical trials [20]. *Callista chione* is the edible bivalve snail, scientifically categorized under the family Veneridae, and is mostly found in marine habitats, in sandy or muddy bottoms. Because of its mild, slightly salty flavor, it is widely used in seafood dishes in many countries.

Many bioactive compounds originate from marine organisms like sponges, mollusks, algae, and bivalves, with the Veneridae family, including the smooth clam *Callista chione*, are notably abundant in Timsah Lake, Ismailia, Egypt [12]. Despite the absence of prior research on the effectiveness of small bioactive peptides isolated from the bivalve *C. chione*, it remains a promising source for such peptides with potential therapeutic benefits.

Therefore, this study represents the first evidence of isolating and characterizing novel peptides from the edible bivalve *C. chione*. Furthermore, this study assessed the safety of these promising peptides on normal human hepatocytes and VERO cell lines on one hand, while investigating the cytotoxic effects on the human HCC cell line (HEPG2) and CRC cell line (HT29) on the other hand.

## 2 Materials and methods

### 2.1 Collection of bivalve *Callista chione*

*Callista chione* is among the most commonly consumed bivalves by the local population of the Suez Canal region. Samples were collected from two stations in Lake Timsah, namely Etab and the Presidential Resthouse, at depths ranging from 3 to 7 m. Lake Timsah is situated 76 kilometers south of Port Said and approximately halfway along the Suez Canal, covering a surface area of 14 square kilometers. Specimen sizes ranged between 2.0 and 3.0 centimeters in shell length. Following collection, they were transferred to a flow-through aquarium system before being transported to the laboratory for dissection and collection of soft tissue.

### 2.2 Total peptides isolation

Soft tissues were dissected from bivalves and ground in a mortar with an ice-cold precipitation solution containing 24 % Trichloroacetic acid (TCA) and 0.07 % β-mercaptoethanol (β-ME) in acetone. The mixture was then immersed in ice and briefly homogenized with a vortex. The homogenized samples were placed in a freezer at −20 °C for 2 h. Centrifugation at 21,000 xg at 4 °C for 30 minutes was used to remove the insoluble supernatant. The resulting pellet was washed twice with 10 ml of ice-cold solution (0.07 % β-ME in acetone) at 2-hour intervals, keeping the temperature at −20 °C. Finally, the pellet containing total peptides was dissolved in 50 mM tris base pH 8. [38].

### 2.3 Purification of peptides by FPLC chromatographic method

The crude peptides extract was concentrated using vivaspin 20 (3 kDa MWCO) filtration system (Sartorius, USA) as a partial purification step of peptides by eliminating small molecules and impurities, thereby improving the quality of the peptides sample for further purification steps. The peptide mixture’s concentration was detected using NanoDrop 2000 at the absorbance of 210 nm. The mixture was purified using fast-performance liquid chromatography (FPLC, AKTA purifier 100) 1 ml SPFF strong cation exchange column (GE Healthcare Life Sciences USA). Firstly, the column was washed with two column volumes (CV) of the start buffer (50 mM tris base pH 8) followed by a linear isocratic elution was carried out by 20 CV with a mixture of start buffer and elution buffer (50 mM tris base pH 8, 1 M NaCl). Five fractions at the absorbance of 210 nm were separated and collected. The fractions’ concentrations were measured using NanoDrop 2000 (OD: 210 nm) [26].

### 2.4 Cell lines and cultures

Herein we chose the commonly used cell lines in toxicological studies to assess the general safety of bioactive compounds to evaluate any potential cytotoxicity on non-cancerous cells. Normal human hepatocytes (ATCC: CRL-2706) and normal kidney epithelial cells taken from an African green monkey (VERO cells, ATCC: CCL-81) were used to test the safety of five isolated fractions. Nonetheless, the anticancer activity of all fractions was assessed against HepG2 (ATCC: HB-8065) and HT-29 (ATCC: HTB-38) cell lines. The cell lines were obtained from the VACSERA Tissue Culture Unit in Dokki, Giza, Egypt. All cells were proliferated in Dulbecco's Modified Eagle's medium (DMEM; Lonza, Walkersville, MD, USA) accompanied by 10 % heat-inactivated fetal bovine serum (FBS) (Gibco, Life Technology, USA) and 1 % L-glutamine, 4-(2-hydroxyethyl)-1-piperazineethanesulfonic acid (HEPES) buffer, 100 U/ml penicillin, and 50 μg/ml gentamycin (Gibco, Life Technology, USA). The cells were maintained in an incubator set at 37°C with a controlled humidified atmosphere containing 5 % CO2. Subculturing was performed twice a week, and cells were passaged upon reaching 80 % confluence.

### 2.5 Safety and cytotoxicity assay

Utilizing the MTT assay (3-(4,5-dimethylthiazol-2-yl)-2,5-diphenyltetrazolium bromide), all fractions were assessed for growth inhibition. One x 10^4 cells per well of 96-well plates was used to seed the cells, which were then incubated over the entire night. The next day, the media were taken out and the cells were treated with various doses of each fraction (2.5–80 μg/ml) or vehicle control for 24 h. Following incubation, each well was filled with 10 % MTT solution (2 mg/ml, Sigma Aldrich, USA), and the cells were left to incubate for an additional 4 h at 37°C in dark conditions. After that, for five minutes, formazan crystals were constantly shaken in 100 μl of dimethyl sulfoxide (DMSO) in each well (Sigma Aldrich, USA). A Bio Tek microplate reader from Agilent Technologies, USA was used to detect absorbance at 540 nm. Equation [1— (ODt/ODc) × 100 %] was used to determine the vitality of the cells, where ODt represents the optical density of the sample-treated wells and ODc represents the mean optical density of the untreated cells. Technical triplicates of each treatment were evaluated, and biological triplicates of the tests were carried out. IC_50_ values were then calculated for every fraction. [30]

### 2.6 Morphology study

Following treatment of each cell line with DMSO or different concentrations of each fraction, cells were cultured in 24-well plates for 24 h. Then, using an inverted light microscope (Optika, Italy), the treated cells were examined and contrasted with the untreated cells, and representative images were taken for further analysis. [22]

### 2.7 Peptide identification using mass spectrometry

The most biologically safe with the highest potential anticancer fractions were analyzed using an Orbitrap Exploris 480 mass spectrometer (Thermo Scientific). At the first bioactive peptides’ fractions were subjected to UHPLC, Buffer A was aqueous formic acid (0.05 %) and Buffer B was formic acid (0.05 %) in acetonitrile [24]. Each sample was injected into a trapping column (C18, 5 µm particle size, Thermo Scientific) and washed with a gradient flux of 98 % Buffer A and 2 % Buffer B at a flow rate of 0.3 ml/min for 20 min, 60 % Buffer A and 40 % Buffer B at a flow rate of 0.3 ml/min for 2 min, 20 % Buffer A and 80 % Buffer B at a flow rate of 0.3 ml/min for 2 min, 98 % Buffer A and 2 % Buffer B at a flow rate of 0.3 ml/min for 6 min. The MS was operated as follows:

i. MS full scan
  Ion Source Type: H-ESI, Spray Voltage: Static, Positive Ion (V): 3800, Negative Ion (V): 3000, Gas Mode: Static, Sheath Gas (Arb): 25, Aux Gas (Arb): 10, Sweep Gas (Arb): 0, Ion Transfer Tube Temp (°C): 320, Vaporizer Temp (°C): 150, Default Charge State: 2, Master Scan: Full Scan, Orbitrap Resolution: 60000, Scan Range (*m/z*): 200–2000, RF Lens (%): 40, Normalized AGC Target (%): 300, Polarity: Positive, Intensity Threshold: 1.0^e3^.
ii. ddMS^2^

Isolation Window (*m/z*): 2, Collision Energy Type: Normalized, HCD Collision Energies (%): 30, Orbitrap Resolution: 15000, Normalized AGC Target (%): 100.

### 2.8 Denovo peptides sequencing and analysis

According to the protein database (www.uniport.org), the full proteome for *Callista chione* bivalve was not available and there were limited sequences for specific proteins, as shown in Table 1. Peptide analysis was performed for the target peptide using Biopharma Finder software 4.0 (Thermo Scientific), *de novo* sequencing using novor.cloud proteomics mass spectrometry data analysis web tool (https://app.novor.cloud/).

**Table 1:** Identified *Callista chione* snail proteins from UniProt database.

**Entry**	**Entry Name**	**Protein names** **EC 7.1.1.9**	**Gene Names**	**Length**
A0A172MLM3	A0A172MLM3_9BIVA	Cytochrome c oxidase subunit 1	CO1	157
A0A172MLW0	A0A172MLW0_9BIVA	Cytochrome c oxidase subunit 1	CO1	157
A0A172MLX0	A0A172MLX0_9BIVA	Cytochrome c oxidase subunit 1	CO1	157
Q0QBQ9	Q0QBQ9_9BIVA	Cytochrome c oxidase subunit 1	COI	190
V9P7E1	V9P7E1_9BIVA	Cytochrome c oxidase subunit 1	COX1	166
Q0QBM9	Q0QBM9_9BIVA	Histone H3		109

The sequences of peptides obtained from Novor.cloud were submitted to the antiCP 2.0 web server (https://webs.iiitd.edu.in/raghava/anticp2/). A prediction score for the effectiveness of antiCP 2 was generated for every submitted peptide by the strict antiCP mechanism. Scores scored from the in silico experiments showed a programmable range from 0 to 1, and higher scores implied anticancer activity. Peptides with a value above the threshold (0.5) functioned as an anticancer peptide. The data generated by the AntiCP 2 provide insights into the anticancer potential activity of multiple peptide sequences. Physicochemical properties such as molecular weight (MW), hydrophobicity, steric hindrance, side bulk, hydropathicity, amphipathicity, hydrophilicity, net hydrogen content, charge, isoelectric point (pI), and physicochemical properties have also been reported. Through these features, one can know to a great extent the type of every peptide, as well as its chemical and functional properties. [1]

### 2.9 Quantitative analysis using qPCR

Total RNA was isolated from HepG2 and HT-29 cells exposed to IC_50_ of promising bioactive peptides’ fractions containing peptides using Trizol reagent (Biovision Co. LTD, Korea) according to the instructions of the manufacturer. cDNA was produced using the Thermo Scientific RevertAidTM First Strand cDNA Synthesis Kit (catalog number K1621) following the manufacturer's instructions. Table 2 illustrates the primers used for beclin-1, Bcl-2, caspase-3, FGFR4, and FGF19. The housekeeping gene, glyceraldehyde-3-phosphate dehydrogenase (GAPDH), was used for normalization. [29]

**Table 2:** Primer sequences used in quantitative real-time PCR analysis.

Target gene (s)	Primer sequence	Accession No
Beclin−1	Forward primer: 5’- GAGAGACCCAGGAGGAAG −3’ Reverse primer: 5’- GGCCCGACATGATGTCAA −3’	XM_017025264.3
Bcl−2	Forward primer: 5’- CCTGGCTGTCTCTGAAGACC −3’ Reverse primer: 5’- CTCACTTGTGGCCCAGGTAT −3’	NM_016993.2
Caspase−3	Forward primer: 5’- TGCATACTCCACAGCACCTG −3’ Reverse primer: 5’- TCTGTTGCCACCTTTCGGTT −3’	XM_054350958.1
FGFR4	Forward primer: 5’- CACTGGTACAAGGAGGGCAG −3’ Reverse primer: 5’- ATCGTTGCTGGAGGTCAAGG −3’	NM_001354984.2
FGF19	Forward primer: 5’- TGTGTGGTGGTCCACGTATG- 3’ Reverse primer: 5’- CGGATCTCCTCCTCGAAAGC- 3’	NM_005117.3
GAPDH	Forward primer: 5’- CCCATCACCATCTTCCAGGAGC −3’ Reverse primer: 5’- CCAGTGAGCTTCCCGTTCAGC−3’	NM_001357943.2

### 2.10 The colorimetric ELISA assay for caspase-9 and p-AKT

HepG2 and HT-29 cells (1 × 10^4^) were exposed to IC_50_ of fractions 4 and 5 and concentrations of cleaved caspase-9 and p-AKT were detected according to the manufacturer’s procedures (SunRed, PELOBIOTECH GmbH, Germany).

### 2.11 Western blot to detect the expression of caspase 3 and Bcl-2

A Western blotting approach was used to assess the Bcl-2 and caspase-3 protein expression levels. We generated cell lysates by subjecting HepG2 and HT-29 cells to the IC_50_ concentrations of fractions 4 and 5 for a full day. To extract proteins, these lysates were centrifuged for 10 minutes at 13,000 g and 4°C. The Bradford assay [27,28] was used to measure the quantities of proteins. We next used 10 % SDS-PAGE to fractionate 20 µg of proteins per lane, which we then put onto nitrocellulose membranes. The transfer was conducted at 80 volts for 2 h. After the transfer, the nitrocellulose membranes were blocked for an hour with shaking in TBST containing 3 % BSA. They were then incubated with primary antibodies against β-actin (Abcam, USA, Cat. No. GTX109639) as a loading control, Bcl-2 (Abcam, USA, Cat. No. GTX100064), and caspase-3 (Abcam, USA, Cat. No. GTX110543) for an additional night at 4°C. The membranes were then treated for two h at room temperature on a rotating shaker with the appropriate secondary antibodies that were conjugated to horseradish peroxidase (HRP) (Antirabbit IgG alkaline phosphatase conjugate, Sigma Aldrich, USA, Cat. No. A3812). Following membrane washing, blots were scanned, and the levels of protein expression were compared to β-actin. Densitometric analysis was utilized to quantify the bands. [34]

### 2.12 Cytopathological examinations

Bioactive peptides’ fractions 4 and 5 were treated at IC_50_ concentrations on HepG2 and HT-29 cells (1 × 10^4). Following treatment, the cells were collected in a tube, trypsinized, and rinsed with PBS (pH = 7.4). The obtained bioactive peptides’ fractions were subsequently centrifuged using a Shandon Cytospin (Thermo Fisher Scientific, Waltham, Massachusetts) for 15 minutes at 1500 rpm. The resultant cell pellet was placed on glass slides and fixed in 95 % ethanol for a full day. Hematoxylin and eosin (H & E) staining was applied to the slides after fixation. [22]

### 2.13 Statistical analysis

The data is shown as mean±SD. The software GraphPad Prism 6 (GraphPad Software Inc., San Diego, CA, USA) was used for every statistical analysis. The Student's t-test was employed to assess differences in changes between the groups and the respective control. Asterisks denote the *p*-values: *p* < 0.05 (*), *p* < 0.01 (**), and *p* < 0.001 (***). The dose-response curve graph plots for each concentration were used to determine the half-maximal inhibitory concentration (IC_50_).

## 3 Results

### 3.1 Purification of peptides by FPLC chromatographic method

**Fig. 1:**
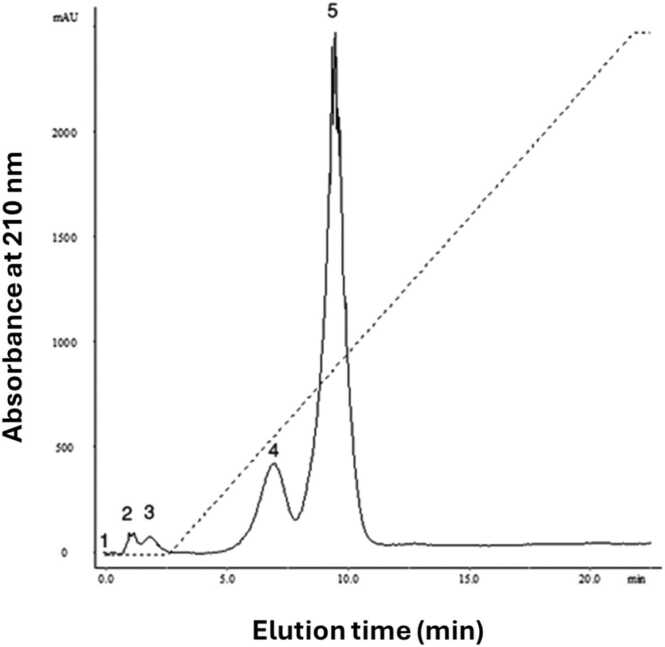
Chromatographic purification of peptides mixture using SPFF cation exchange column and linear isocratic elution. X-axis represents the absorbance at 210 nm and Y-axis represents time in min. The scattered line is corresponding to isocratic elution from 1 % to 100 %.

The concentration of mixed peptides (measured at OD_210_) injected into the column for purification was 5 mg/ml. The peptides were fractionated along the isocratic elution according to their charges. As shown in the chromatogram of Fig. 1, five peaks in elution were observed at retention times 0.21, 0.95, 1.63, 7.65, and 10.34 min, respectively.

The collected fractions were named 1, 2, 3, 4, and 5. Each fraction contained a mixture of peptides with relatively similar charges. The concentration of each fraction was detected to be 0.95 ± 0.25, 9.8 ± .69, 16.32 ± 1.2, 44.8 ± 1.1, and 240.1 ± 1.85 µg/ml respectively at 210 nm. The fractions with high contents of peptides (4 and 5) were subjected to MS analyses to detect the sequences of peptides.

### 3.2 Peptide identification using mass spectrometry

Peptide mapping analysis for the collected fractions 4 and 5 was carried out due to their high concentrations and/or high anticancer efficacy. Analysis was firstly performed using Thermo Biopharma Finder 3.2 and Thermo Proteome Discoverer software but no results were obtained due to the absence of similar *C. chione* protein sequences from databases (NCBI and UniProt). Hence, *de novo* peptide sequencing of fractions 4 and 5 was performed using novor.cloud web tool (https://app.novor.cloud) concerning the SwissProt and NCBI databases. Results showed the identification of 246 peptides (from 5975.061 to 3000.186 Da) in fraction 4 **(**Table S1**)**. Furthermore, peptide mapping analysis on fraction 5 showed that 199 peptides (from 6471.897 to 3001.099 Da) were recognized **(**Table S2**)**.

### 3.3 Analysis of de novo sequencing peptides using AntiCP 2.0

The results of fraction 4 indicated that 119 peptides were classified as non-anticancer peptides while 127 were categorized as anticancer peptides. The anticancer scores for these peptides ranged from a minimum of 0.05 to a maximum of 0.91 and the mean score of 127 anticancer peptides was found to be 0.62 ± 0.1. In terms of physicochemical properties, the maximum hydrophobicity recorded was 0.14, and the maximum hydrophilicity was 1.75. The mean isoelectric point (pI) was 8.3 ± 2.99.

The analysis of fraction 5 showed that 74 peptides were identified as non-anticancer peptides and 125 as anticancer peptides. The anticancer scores in this sample ranged from a minimum of 0.12 to a maximum of 0.89 and the mean score of 74 anticancer peptides was found to be 0.65 ± 0.095. The maximum hydrophobicity observed was 0.18, and the maximum hydrophilicity was 1.76. The mean pI for these peptides was 8.04 ± 2.99.

### 3.4 Cytotoxic activity

**Fig. 2:**
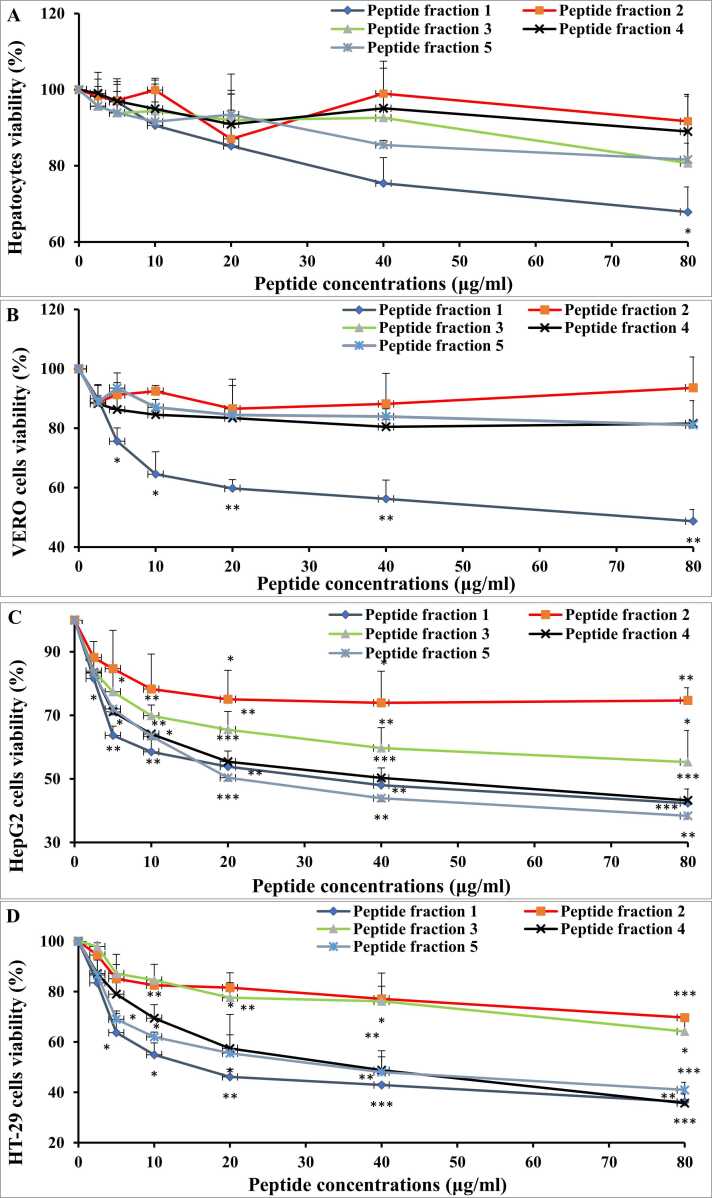
The safety and cytotoxic effect of isolated fractions on (A) human hepatocytes, (B) VERO, (C) HepG2, and (D) HT-29 cells. Values as presented as mean±SD. Student’s *t*-test was used to compare each group with the corresponding control at *P* < 0.05 (*), *P* < 0.01 (**), *P* < 0.001 (***).

Fig. 2 shows the safety and cytotoxicity results of the five fractions isolated from *C. chione* against normal cells (normal human hepatocytes and VERO cell lines) and tumor cells (HepG2 and HT-29). The cell viability of normal human hepatocytes and VERO cell lines remained unchanged significantly from the control group for all fractions, except for fraction 1 at 80 µg/ml in hepatocytes and 5, 10, 20, 40, and 80 for VERO cells.

Fractions 4 and 5 were confirmed to be safe on normal hepatocytes and exhibited the highest cytotoxic activity against the HepG2 cell line, with IC_50_ values of 28.99 and 23.39 µg/ml, respectively. Similarly, fractions 4 and 5 have shown maximum significant antitumor potential against HT29 cells with IC_50_ values of 30.45 μg/ml and 26.72 μg/ml, respectively. Concerning their safety to VERO cells, The IC_50_ values of the other peptides were found to be outside the tested concentration range. Specifically, their IC_50_ values exceeded the highest tested concentration (80 μg/ml) against both HepG2 and HT-29 cell lines after 24 h of incubation. Based on the safety profile and IC_50_ values, only fractions 4 and 5 demonstrated significant inhibitory activity, making them the most promising among the five fractions. Table 3

**Table 3:** IC_50_ values of fractions on normal and cancer cells.

**Cancer Cells/Fraction**	**Fraction 4**	**Fraction 5**
**HepG2**	28.99 μg/ml (4.48 μM)	23.39 μg/ml (7.79 μM)
**HT29**	30.45 μg/ml (4.70 μM)	26.72 μg/ml (8.90 μM)

### 3.5 Morphology study

**Fig. 3:**
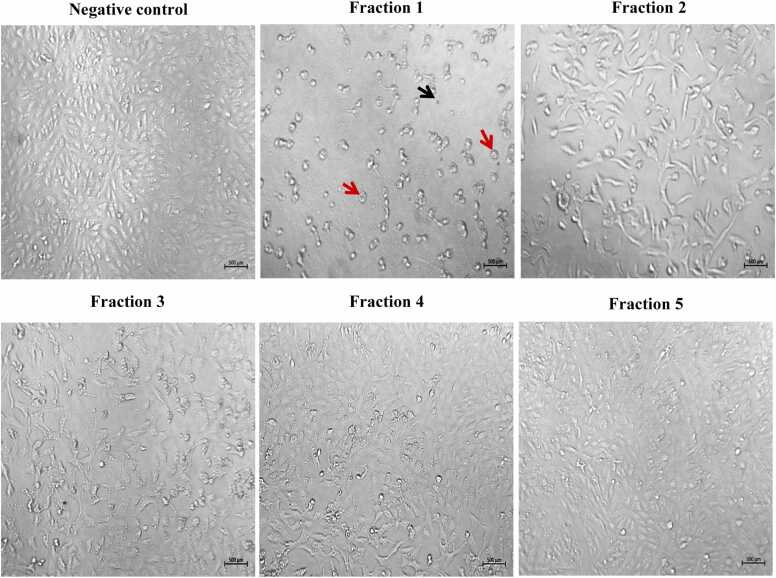
The effect of different fractions (concentration 80 μg/ml) on cell morphology of normal human hepatocytes cell line after 24 h of incubation. Images were captured under a brightfield microscope. Apoptotic cells are marked by black arrows. Rounded cells are marked by red arrows (500x).

**Fig. 4:**
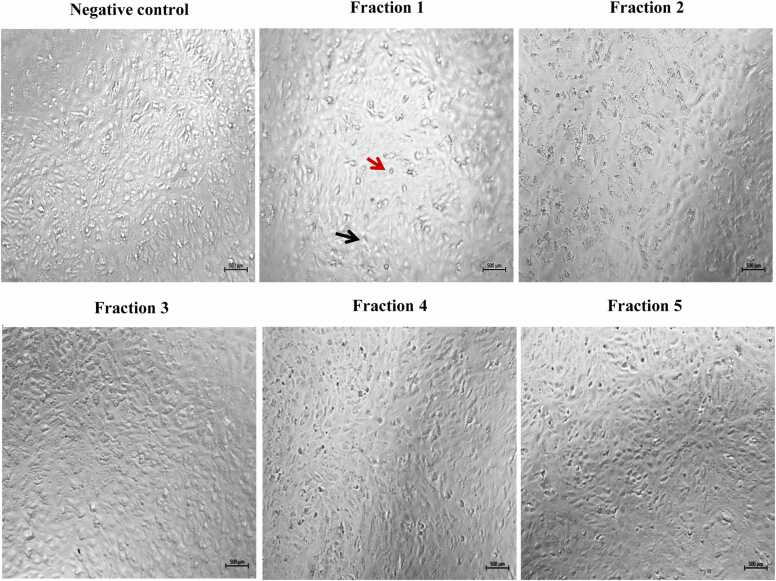
The effect of different fractions (concentration 80 μg/ml) on cell morphology of VERO cell line after 24 h of incubation. Images of cells were captured under a brightfield microscope. Apoptotic cells are marked by black arrows. Rounded cells are marked by red arrows (500x).

**Fig. 5:**
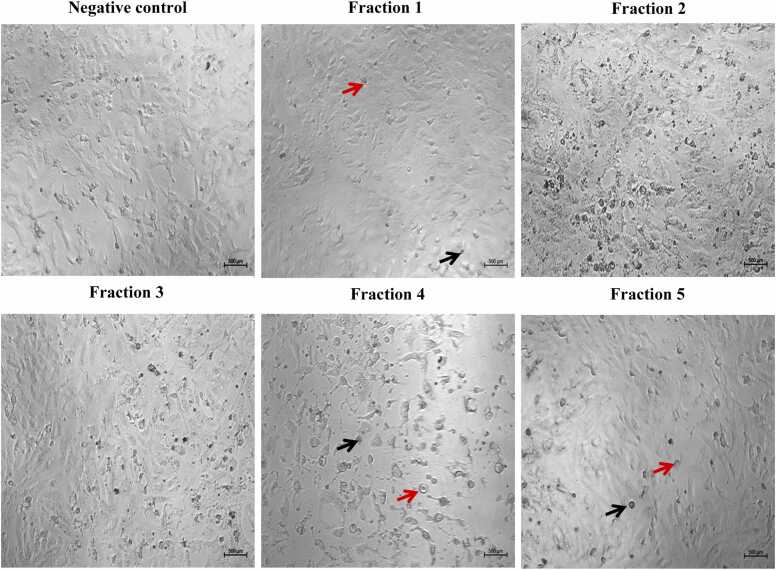
The effect of different fractions (concentration 80 μg/ml) on cell morphology of HepG2 cell line after 24 h incubation. Images of cells were captured under a brightfield microscope. Apoptotic cells are marked by black arrows. Rounded cells are marked by red arrows (500x).

To investigate the impact of the isolated peptides on tumor cell growth, morphological changes in both normal and malignant cells were examined using an inverted light microscope. After 24-hour treatment with the highest concentrations of various fractions, it was observed that normal human hepatocytes and VERO cell lines showed a decrease in cell density and noticeable morphological alterations specifically in cells treated with bioactive fraction 1. This indicates the potential toxicity of fraction 1 in normal cell lines **(**Fig. 3 **and** Fig. 4**)**. The treated HepG2 cells demonstrated a decline in cell counts upon exposure to fractions 1, 4, and 5. Specifically, HepG2 cells exhibited a rounded morphology with a significant decrease in both cell number and size. Moreover, these treated cells exhibited clear signs of apoptosis, including loss of contact with neighboring cells, formation of apoptotic bodies, cellular shrinkage, detachment from the culture plate, and cell fragmentation, when compared to their untreated counterparts **(**Fig. 5**)**.

**Fig. 6:**
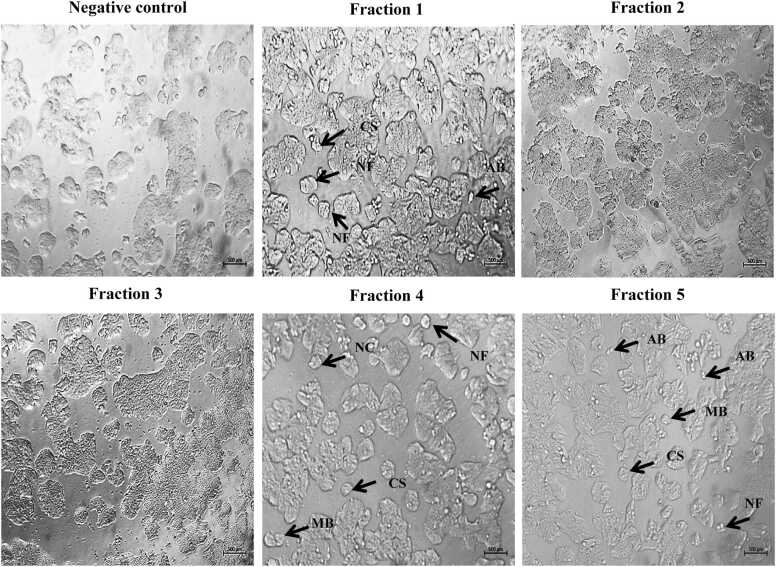
The effect of different fraction (concentration 80 μg/ml) on cell morphology of HT-29 cell line for 24 h and images of cells were captured under a brightfield microscope. Characteristic of apoptosis such as nuclear compaction (NC), apoptotic bodies (AB), membrane blebbing (MB), cellular shrinkage (CS), and nuclear fragmentation (NF) were illustrated (500 ×).

Furthermore, HT-29 cells treated with fractions 1, 4, and 5 displayed altered morphology characterized by reduced cell size, a rounded shape, and nuclear compaction. Additionally, these treated cells exhibited apoptotic features such as the presence of apoptotic bodies, membrane blebbing, cellular shrinkage, and nuclear fragmentation **(**Fig. 6**)**.

### 3.6 Effect of IC_50_ of fractions 4 and 5 on apoptotic and autophagy biomarkers

**Fig. 7:**
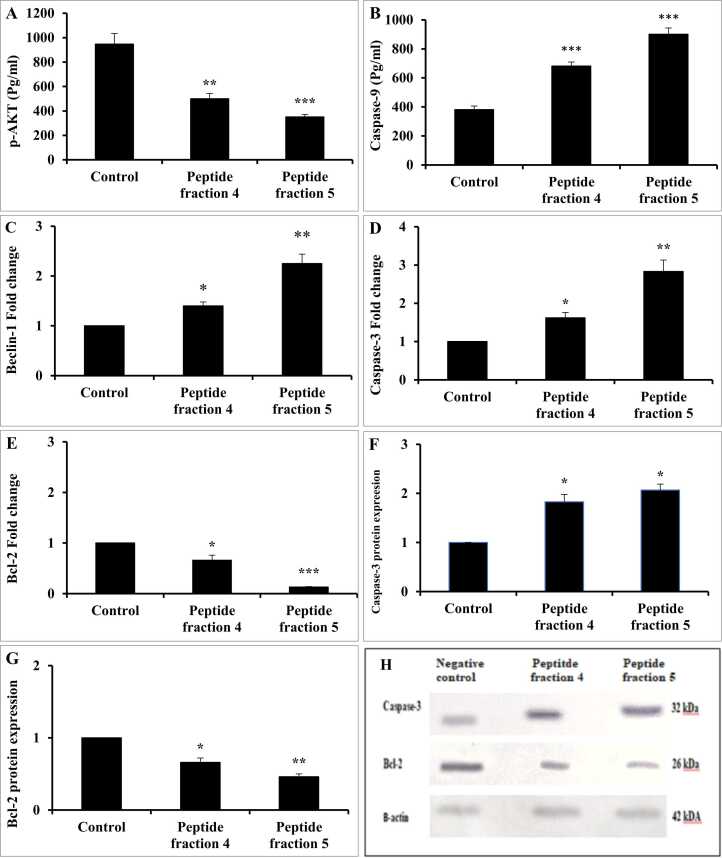
The effect of fractions 4 & 5 in HepG2 cells on (A) p-AKT and (B) caspase-9 contents as well as (C) beclin-1, (D) caspase-3, and (E) Bcl-2 gene expressions and (F) caspase-3 protein expression, (G) Bcl-2 protein expression and (H) western blot image of caspase-3 and Bcl-2. Values as presented as mean±SD. Student’s *t*-test was used to compare each group with the corresponding control at *P* < 0.05 (*), *P* < 0.01 (**), *P* < 0.001 (***).

**Fig. 8:**
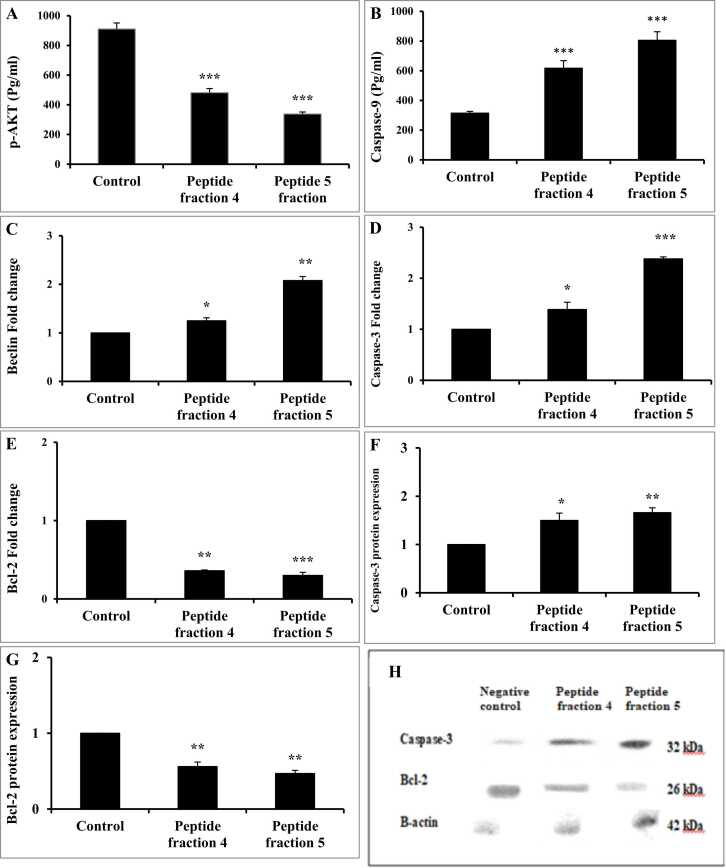
The effect of fractions 4 & 5 in HT-29 cells on (A) p-AKT and (B) caspase-9 contents as well as (C) beclin-1, (D) caspase-3, and (E) Bcl-2 gene expressions and (F) caspase-3 protein expression, (G) Bcl-2 protein expression and (H) western blot image of caspase-3 and Bcl-2. Values as presented as mean±SD. Student’s *t*-test was used to compare each group and the corresponding control at *P* < 0.05 (*), *P* < 0.01 (**), *P* < 0.001 (***).

The results revealed that p-AKT content was significantly decreased in HepG2 cells by 47.18 % and 62.91 % after exposure to fractions 4 and 5, respectively when compared to control untreated cells **(**Fig. 7**A)**. Similarly, p-AKT content was significantly diminished in HT-29 cells by 47.07 % and 62.93 % after exposure to fractions 4 and 5, respectively in relation to the control untreated cells **(**Fig. 8**A)**. Caspase-9 contents were significantly increased in HepG2 cells by 78.72 % and 136.44 % after exposure to fractions 4 and 5, respectively when compared with control untreated cells **(**Fig. 7**B)**. Similarly, caspase-9 content was significantly elevated in HT-29 cells by 96.46 % and 156.18 % after exposure to fractions 4 and 5, respectively in relation to the control untreated cells **(**Fig. 8**B)**. In addition, Beclin-1 gene expression level was found to be up-regulated upon treatment of HepG2 with fractions 4 and 5 by 1.4 and 2.25 folds, respectively when compared to the control group **(**Fig. 7**C)**, while the analogous level in HT-29 was found to be up-regulated by 1.25 and 2.08 folds, respectively (*p* < 0.05 and *p* < 0.01, respectively) in comparison to the control group **(**Fig. 8**C)**. Furthermore, the expression of the pro-apoptotic caspase-3 gene and its protein was significantly increased in HepG2 cells **(**Fig. 7**D, F)** and HT-29 cells **(**Fig. 8**D, F)** after exposure to fractions 4 and 5, compared to the corresponding negative control. Additionally, the expression of the anti-apoptotic Bcl-2 gene and its protein was significantly decreased in HepG2 cells **(**Fig. 7**E, G)**, with a similar decrease observed in HT-29 cells **(**Fig. 8**E, G),** following treatment with fractions 4 and 5, compared to the corresponding negative control.

### 3.7 Influence of IC_50_ of fractions 4 and 5 on FGFR4 and FGF19 gene expression levels

**Fig. 9:**
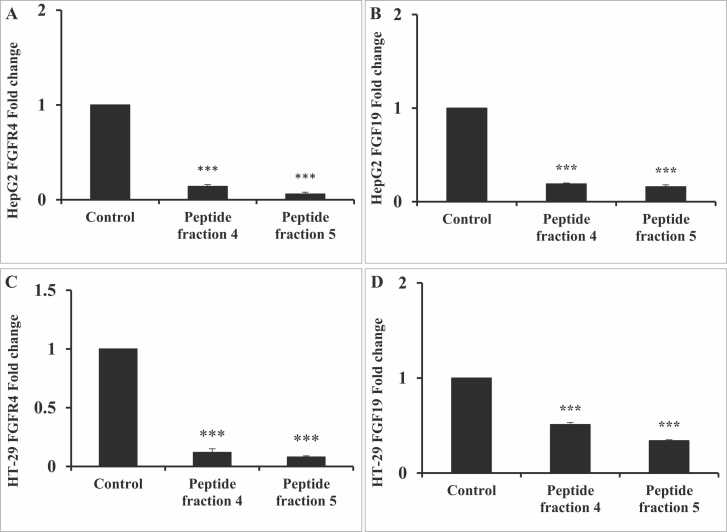
The effect of exposure to IC_50_ fractions 4 & 5 for 24 h on (A) HepG2 FGFR4, (B) HepG2 FGF19, (C) HT-29 FGFR4, (D) HT-29 FGF19 genes expression. Data are presented as mean±SD. Student’s *t*-test was used to compare each group with the corresponding control at *P* < 0.001 (***).

In Fig. 9, our data illustrates a significant decrease (P < 0.001) in the gene expression levels of FGFR4 and FGF19 in HepG2 cells when compared to the control group. Specifically, after exposure to fraction 4, the expression levels decreased by 86 % and 81 %, respectively, whereas exposure to fraction 5 resulted in a decrease of 94 % and 92 %, respectively, compared to untreated control cells. Similarly, in HT-29 cells, a significant decrease (P < 0.001) in FGFR4 and FGF19 gene expression levels was observed. Specifically, exposure to fraction 4 has decreased the expression levels of FGFR4 and FGF19 by 88 % and 49 %, respectively, while exposure to fraction 5 led to a decrease of 92 % and 66 %, respectively, compared to control untreated cells.

### 3.8 Effect of fractions 4 and 5 on cytopathological examinations

**Fig. 10:**
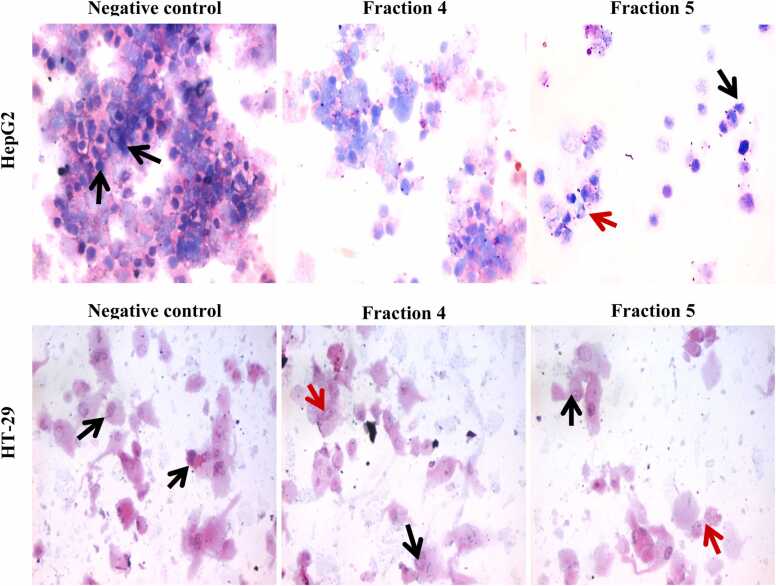
Cytospin smear stained by H & E after exposure HepG2 and HT-29 with IC_50_ of fractions 4 & 5. Black arrow indicates nucleocytoplasmic ratio. Red arrow indicatesa the scattered apoptotic and degenerative changes (400x).

Treatment of HepG2 and HT-29 cells with IC_50_ of fractions 4 and 5 had an impact on the morphology and characteristics of the cells. The findings from Fig. 10 indicate that untreated HepG2 cells displayed a significant presence of neoplastic hepatocytes, marked by clusters of cells showing enlarged nuclei and an elevated nucleocytoplasmic ratio. Comparably, groupings of epithelial cells with longer nuclei and a greater nucleocytoplasmic ratio were among the numerous neoplastic cancer cells observed in untreated HT-29 cells. On the other hand, HepG2 cells treated with fractions 4 and 5 at IC_50_ values showed a dispersed presence of centrally nucleated hepatocytes along with dispersed degenerative and apoptotic alterations. Furthermore, HT-29 cells treated with the IC_50_ concentrations of peptides 4 and 5 fractions demonstrated a moderate number of colon carcinoma cells with central nuclei and a moderate occurrence of apoptotic and degenerative changes.

## 4 Discussion

Chemotherapy continues to play a pivotal role in cancer treatment [25], yet its side effects present considerable hurdles [6]. Consequently, there has been increasing interest in exploring chemo-preventive approaches, employing both natural and synthetic drugs, to alleviate the burden of disease. Recently, significant investigation attempts have been directed at investigating the preventative and treatment potentials of various natural compounds and dietary supplements targeting specific cancer types [8]. Notably, the emergence of marine bioactive peptides represents a burgeoning area in drug development. Thriving in unique environments characterized by hyper-salinity, high pressure, hypoxia, hypothermia, and sunlight deprivation, marine organisms produce proteins and peptides with distinct bioactivities and structural features (S. [40]). Marine peptides possess a versatile range of properties, including immunomodulatory, tissue regeneration, antioxidant, antimicrobial, antiviral, cardioprotective, and antitumor effects [23]. Unlike conventional chemotherapy agents, small peptides offer several advantages such as improved absorption, compact size, precise targeting, and reduced toxicity [14], making them a promising avenue for investigation. Additionally, their simple peptide linkages and low molecular weight make them conducive to modification and synthesis, further highlighting their potential in biomedical research.

This study represents a pioneering effort in isolating and characterizing novel peptides derived from *C. chione*. The focus of the study is on purifying and identifying fractions and then evaluating them for anticancer potentials. The mixed peptides were purified using FPLC, during that time five separate fractions were collected based on their retention time. The concentration for each fraction was detected, which showed a different intensity of peptides for each fraction, with the highest intensity in the last fraction. The identified peptides in fractions 4 and 5 using novor.cloud tool were then analyzed using the AntiCP 2.0 tool to evaluate their anticancer potential and physicochemical properties. The total anticancer score is computed from the bioactivity predictions of machine learning where each peptide is assigned a score ranging between 0 and 1 for the probability of exhibiting anticancer activity based on the physicochemical properties and the bioactivity of similar peptides is applied to score and measure the anticancer properties of isolated peptides. Positive selection of probable anticancer peptides was based on a rating ≥ 0.5 to allow screening of promising candidates. Solubility is another parameter; calculated from hydrophilicity with a mean result of 1.75, this property increases the solubility and stability of the peptides, raising their bioavailability along with possible therapeutic outcomes. The results of these tools for the two fractions 4 and 5 concluded that the total anticancer score mean of peptide mixtures in them was ≥ 0.5 which is above the score threshold thus considering the fractions possess anticancer properties. On the other hand, the hydrophilicity of peptides in the two fractions was 1.75 which is higher than the hydrophobicity which was 0.14–0.18 obtained from AntiCP 2.0 [1], this means that the peptides dissolve or interact more readily with water and aqueous solutions. Hence, the identification of a substantial number of anticancer peptides suggests that the peptides derived from *C. chione* could be promising candidates for further investigation in cancer research. From de novo sequencing, the obtained peptides contain cysteine residues, which form disulfide bonds to further stabilize the structure, thus improving their resistance to degradation which is important for restoring high efficiency and targeting cancer cells. Also, hydrophobic and aromatic groups in addition to amphipathic and repetitive motifs allow for increased interaction with the cell membrane to form stable complexes with the cancer cell membrane, which increases their ability to destabilize a cell and lead to cell death.

Moreover, the safety profile assessment confirmed the safety of these peptides on normal human hepatocytes and VERO cell lines except for fraction 1. Furthermore, the examination of their cytotoxic effects on HEPG2 and HT29 cell lines provided valuable insights into their potential activity as therapeutic agents against these cancer types. Fractions 4 and 5 exhibited the highest levels of cytotoxic activity against the HepG2 and HT-29 cell lines. Notably, peptides 3 and 4 fractions did not display any noticeable cytotoxic effects, even at the highest tested concentration of 80 μg/ml after 24 h of incubation. Based on these findings, peptides 4 and 5 fractions emerge as the most promising candidates for further exploration due to their significant inhibitory effects on HepG2 and HT-29 cells, alongside their demonstrated safety on normal human hepatocytes and VERO cell lines.

We then asked what is the underlying mechanisms that fractions 4 and 5 stimulate to achieve such a cytotoxic effect. Prior research has demonstrated that the absence of apoptosis occurs in various human tumors, potentially resulting in the transformation of a healthy cell into a cancerous one [13]. Moreover, autophagy has emerged as a crucial cell signaling pathway and a significant focus in cancer research. This process is governed by autophagy-related genes (ATG), which are essential for initiating autophagy, forming vesicles, and completing the formation of autophagosomes [10]. The present study investigated the effects of fractions 4 and 5 in HepG2 and HT-29 cancer cell lines through the regulation of autophagy and apoptosis through the interaction between Beclin-1 and Bcl-2 significantly impacts the p-AKT pathway as shown before in different cancer types [21]. The induction of the mitochondrial pathway of apoptosis was evidenced by increased caspase-9 and caspase-3 genes and protein expressions, alongside a significant reduction in Bcl-2 gene and protein expressions as explained recently [5]. These findings match with other recent anticancer studies on HepG2 cell lines [31] and HT-29 [15]. These findings were corroborated by cytospin smears of HepG2 and HT-29 cells treated with IC_50_ concentrations of fractions 4 and 5, which displayed scattered apoptotic and degenerative changes. Additionally, exposure of HepG2 and HT-29 cells to IC_50_ concentrations of these fractions induced autophagy, as indicated by a significant decrease in p-AKT levels and a marked increase in Beclin-1 gene expression as shown before on HepG2 with different treatment [37]. Overall, the study suggests that fractions 4 and 5 may exert anticancer effects through the induction of both apoptosis and autophagy.

FGFs-FGFRs play crucial roles in controlling numerous biological functions like embryonic development, cell proliferation, differentiation, and tissue regeneration. Dysregulation of FGF-FGFR signaling is frequently observed across various diseases, disorders, and cancer types. Particularly, the abnormal expression of FGF19/FGFR4 is implicated in the advancement of HCC [7]. The results of this study revealed that fractions 4 and 5 inhibited the FGF19–FGFR4 pathway, suggesting another important mechanism of their anticancer activity. The inhibition of this pathway, in addition to the induction of apoptosis and autophagy, highlights the multifaceted anticancer effects of these bioactive peptides. Specifically, the suppression of the FGF19–FGFR4 signaling could contribute to the reduction of cancer cell proliferation and survival, further supporting the potential of fractions 4 and 5 as promising anticancer agents.

## 5 Conclusion

In conclusion, this study underscores the promising anticancer potential of peptides sourced from the bivalve *C. chione*, with particular emphasis on fractions 4 and 5. These peptides demonstrated notable cytotoxic effects on HepG2 and HT-29 cancer cell lines, primarily through mechanisms involving the induction of apoptosis and autophagy. The interaction between Beclin-1 and Bcl-2 proteins emerged as a crucial factor, influencing the p-AKT pathway and leading to increased expressions of caspase-9 and caspase-3, alongside a reduction in Bcl-2 levels. Additionally, the peptides inhibited the FGF19–FGFR4 pathway, further enhancing their anticancer activity by suppressing cancer cell proliferation and survival. Importantly, these bioactive peptides exhibited a favorable safety profile, showing no toxicity to normal human hepatocytes and VERO cell lines, except for fraction 1. In conclusion, fractions 4 and 5 of the peptides from *C. chione* emerge as potent and safe candidates warranting further in vivo investigation for cancer therapy development.

## Ethical approval

Ethical approval for the study was obtained from the TBRI Ethics Committee (FWA00010609; PT 738). This work did not involve human subjects, it did not require a clinical trial registration number. The research focused on evaluating the biological activity and safety profile in vitro.

## Funding

The authors received no financial support for the research, authorship, and/or publication of this article.

## CRediT authorship contribution statement

**Hussein Ahmed:** Writing – review & editing, Writing – original draft, Visualization, Validation, Supervision, Software, Resources, Project administration, Methodology, Investigation, Funding acquisition, Formal analysis, Data curation, Conceptualization. **Okasha Hend:** Writing – review & editing, Writing – original draft, Visualization, Validation, Supervision, Software, Resources, Project administration, Methodology, Investigation, Funding acquisition, Formal analysis, Data curation, Conceptualization. **ElZallat Mohamed:** Writing – original draft, Visualization, Investigation, Formal analysis, Data curation. **El-Dabaa Ehab:** Supervision, Software, Project administration, Funding acquisition. **Salem Maha:** Writing – review & editing, Writing – original draft, Visualization, Validation, Supervision, Software, Resources, Project administration, Methodology, Investigation, Funding acquisition, Formal analysis, Data curation, Conceptualization. **Hammam Olfat:** Writing – original draft, Visualization, Resources, Investigation. **Ghoname Samah:** Writing – original draft, Validation, Software, Methodology, Funding acquisition, Data curation. **Habib Mohamed:** Writing – review & editing, Writing – original draft, Visualization, Validation, Supervision, Software, Resources, Project administration, Methodology, Investigation, Funding acquisition, Formal analysis, Data curation, Conceptualization.

## Declaration of Competing Interest

The authors declare that they have no known competing financial interests or personal relationships that could have appeared to influence the work reported in this paper.

## Data Availability

No data was used for the research described in the article.
