# Supplementary material for: Identification and exploration of anticancer activity of novel peptides isolated from the edible bivalve Callista chione in hepatic and colon cancer cell lines
Source: Toxicol Rep. 2025 Jan 27;14:101915. doi: 10.1016/j.toxrep.2025.101915 (PMC11833620; doi:10.1016/j.toxrep.2025.101915)
Supplement: Supplementary file 1 — Supplementary material [file mmc1.docx]

**Table S1.** Analysis of peptides in fraction 4 using novor.cloud

| **No** | **RT** | **mz(data)** | **Z** | **pepMass**  **(denovo)** | **Err**  **(data-denovo)** | **Ppm** | **Peptide** |
| --- | --- | --- | --- | --- | --- | --- | --- |
| 1 | 1405.6 | 1992.687 | 3 | 5975.061 | -0.0233 | -3.9 | DNNEMHCCDCGCCWHMWWYEWQGEFNVSRRKKYCGMDCCCGMHCCYMCG |
| 2 | 1351.9 | 1985.267 | 3 | 5952.858 | -0.0803 | -13.5 | DHMGWMMMDDMYVSMRRTWWYKYRMRRRMMKKKLHKLLKVVMSST |
| 3 | 209.6 | 1983.403 | 3 | 5947.263 | -0.0748 | -12.6 | DAWWWWWWWWWWWWWNEWEEGGWMMMMRWWWWMMMEMM |
| 4 | 871.1 | 1946.307 | 3 | 5835.985 | -0.0847 | -14.5 | DYMEYDDCMMMMMCMDHCCEDMWWWWWWWWWWWWWWAQE |
| 5 | 1408.3 | 1941.488 | 3 | 5821.474 | -0.0333 | -5.7 | DNDQMCHCMECCMCCCCCSCGRQTMSMDYMMCCCCCWHDDDMHCCMMCG |
| 6 | 1407.8 | 1937.773 | 3 | 5810.313 | -0.0158 | -2.7 | DENNMCCGPRRRHLRRRLRRRKWWEGDDCMMCMDMDCCNMCCGYCGM |
| 7 | 1371.1 | 1936.076 | 3 | 5805.161 | 0.0445 | 7.7 | DAHTRMAFQKKKVVRRRKRSPKWWWWFHHLLLLLRRMKMVQYEN |
| 8 | 882.6 | 1931.857 | 3 | 5792.465 | 0.0827 | 14.3 | DAGEWWWWWWFERLAPKWVPEMQMNWWWWWWWWWWMMEE |
| 9 | 1345.3 | 1930.785 | 3 | 5789.32 | 0.0125 | 2.2 | DNNPYMEEMMCMDMWYMMCSCSRKKVLPLHDDCCCCEKKKKKKYSGD |
| 10 | 1352.1 | 1925.505 | 3 | 5773.57 | -0.0767 | -13.3 | DAHTWEGWWMRRDRRPWWWKMMDAGYDWWWSVWRRPREAMMQ |
| 11 | 1412.3 | 1921.518 | 3 | 5761.575 | -0.0419 | -7.3 | MPCMDCCMEEDDYWWNRMMEMDDDCMCMMMDHECCCCADNCCGYMCG |
| 12 | 1399.2 | 1918.445 | 3 | 5752.269 | 0.0454 | 7.9 | DDCHWDCCWYVKKLLLARLRRKGAWNSDMQQHQCCYDCCMDCCEEDC |
| 13 | 1412.5 | 1901.83 | 3 | 5702.534 | -0.0657 | -11.5 | DNDQMEDDMNCCHDNCRFFEGNCCCCSDDDCCCYCCCMECCMEDDSDDH |
| 14 | 1427.5 | 1888.017 | 3 | 5661.098 | -0.0697 | -12.3 | DMMTMDDCPMQCCWWWMWYCRHRWRRRRRMEGYDCCCHMDDD |
| 15 | 1418.7 | 1881.404 | 3 | 5641.27 | -0.079 | -14 | DFMTWYCRHKKKKQVRARRRMCQERMMLMMMDDCCGMHCCMGCM |
| 16 | 1475.1 | 1876.342 | 3 | 5626.056 | -0.0515 | -9.1 | DCSMCCCYMMNCMMMRRLPKWPRRPWWWWMMEYQHDCDMDDD |
| 17 | 1406.9 | 1849.475 | 3 | 5545.453 | -0.0484 | -8.7 | DEEDMDDCMMCCCCCCMRMMYYMMDWFCMDCMGEMMMCDDMGCF |
| 18 | 1430.2 | 1829.807 | 3 | 5486.466 | -0.0674 | -12.3 | DHEEWYCFKLLARRRRNNWMCMECCCCCGCLSRRAGREETKLLAL |
| 19 | 1509.7 | 1817.155 | 3 | 5448.513 | -0.0686 | -12.6 | DMADDDFHRRRRRMKWPPVYYKWYFKRKREGRKMMCCMDDD |
| 20 | 1228.4 | 1803.882 | 3 | 5408.634 | -0.0108 | -2 | DPPDMRAWKKKWWEMMPVPLRLKHTAWWEMMFKNRRNLPDHG |
| 21 | 1377.6 | 1800.543 | 3 | 5398.671 | -0.065 | -12 | DRHRKKKHAKWWWMWDWMLMRDWWWWMDKRLLRPLMKD |
| 22 | 1329.3 | 1796.18 | 3 | 5385.438 | 0.0793 | 14.7 | LRKKKAKKKVSRRVHKWKWWWQQWYCCCCMCMMMMMMMQM |
| 23 | 1498.9 | 1789.498 | 3 | 5365.392 | 0.0802 | 14.9 | KLAKKRRKKLRRRKKKRRMMCMFMVLLCCCCCGCCCCCCCMDDD |
| 24 | 1409.2 | 1775.304 | 3 | 5322.958 | -0.0694 | -13 | DHEEWYCMECSRTRLRLWRRDNYYMCDHDMECCGCNDTMGCM |
| 25 | 1323 | 1771.852 | 3 | 5312.563 | -0.0295 | -5.5 | DAHTWWWWWWWWWFDWMMEERRTTTLLLLLLLLRLMQM |
| 26 | 1272.4 | 1769.846 | 3 | 5306.456 | 0.0586 | 11 | FLRRRLAKFKDKKKKRKKKKLLLRKKKKKRKSLKPVKVVMQM |
| 27 | 1402 | 1755.647 | 3 | 5263.955 | -0.0372 | -7.1 | DAQACMCCHDQTWEGWWLWYYYMNWWWEGLEQSMEDDYMGC |
| 28 | 1335 | 1755.439 | 3 | 5263.306 | -0.0107 | -2 | NYMYCGCCCCCCCCDDVSRPAYRRRRFDMRKKKKHRKQLYGW |
| 29 | 1456.7 | 1755.24 | 3 | 5262.685 | 0.0144 | 2.7 | WLWAYKPKYRRRRMRMMMMNSMMMDKKVLKLLLVWHKLH |
| 30 | 1388 | 1749.894 | 3 | 5246.655 | 0.0058 | 1.1 | DHNQDAWRRPGASFFWKKKKRRRFTYVWWRRRRFFMDDD |
| 31 | 1022.3 | 1747.169 | 3 | 5238.561 | -0.0748 | -14.3 | DAMRRRRRMWMEDDKKVVDPPDMDRCCKLLALWEGWRRFKP |
| 32 | 1433.3 | 1745.354 | 3 | 5232.966 | 0.0747 | 14.3 | WLAKLLLLLLRKKKPMDCCWHHCRPMRCRKKKVAKKKKLWGF |
| 33 | 1326.4 | 1741.903 | 3 | 5222.654 | 0.0338 | 6.5 | DHDGMHNCEENMMCDMKSDWYDNCKCDMMMQCHCCMAGPEMMQ |
| 34 | 991.9 | 1726.35 | 3 | 5175.956 | 0.0708 | 13.7 | LKVLKKHRRRRKKKKKLWYFEFCWRRVGRRRMERWGPY |
| 35 | 1319.8 | 1712.868 | 3 | 5135.604 | -0.0225 | -4.4 | FNMWWVSKRRAWEEDRLLLLAWPVVSCNRWWKKRREMMQ |
| 36 | 1373.1 | 1711.23 | 3 | 5130.643 | 0.024 | 4.7 | DPPDLGFHRHPWWKKGPKWWMRLPNALCRKKMMRKQLGFMQ |
| 37 | 1500.6 | 1686.871 | 3 | 5057.665 | -0.0732 | -14.5 | DMETWMHWWWWWNVWWTSEEYVMCCCCCCGSMCCMMDDD |
| 38 | 1022.8 | 1674.731 | 3 | 5021.246 | -0.0738 | -14.7 | DACCMDAYEWMFMRHRRRHKKRRRRRRRKYMCCCCSSC |
| 39 | 1348.3 | 1648.214 | 3 | 4941.688 | -0.0683 | -13.8 | DAHTMMCCDMMKCMMMDGWWWWMMMYSENNWTYLVGMC |
| 40 | 1362.3 | 1633.055 | 3 | 4896.205 | -0.0606 | -12.4 | DMGHYFMMMMMMYWWWWRRRFKRKKKYQRGKMMQ |
| 41 | 1395 | 1621.174 | 3 | 4860.568 | -0.0668 | -13.7 | DRCNMCHCMDDCWLWWMHRRRDCCCCGMDDCMCCHYCGM |
| 42 | 1346.5 | 1609.385 | 3 | 4825.181 | -0.0465 | -9.6 | DHRYCYRMMCCCQWMMMQFFRRFKLSVKKKKMLYNE |
| 43 | 510.5 | 1608.352 | 3 | 4821.979 | 0.0537 | 11.1 | DHAWWWQDHVWWWHYMMMMMMRFGGERMRDWRQQ |
| 44 | 1067.6 | 1608.122 | 3 | 4821.283 | 0.0624 | 12.9 | KLLARRVGKSLRKKKKKRKKKLSALKKKKKLLLLPRRVKM |
| 45 | 1070 | 1608.071 | 3 | 4821.127 | 0.0643 | 13.3 | QLRQWWWWWAQMMNWWWWWWRDYYYQMMHPKK |
| 46 | 1350.6 | 1594.456 | 3 | 4780.296 | 0.0496 | 10.4 | DAHTYYNMEEWHHFGKWWMEKLHVKKVLRTTTKLMMQ |
| 47 | 1349.6 | 1593.195 | 3 | 4776.626 | -0.0619 | -13 | DNPNWWWYYCCMRRKKKKLRRRTKKKKKKRKVYTGT |
| 48 | 1409 | 1586.435 | 3 | 4756.351 | -0.0681 | -14.3 | DDNQFMCDMMDCCCCWWMHCSWWCMPEMMMCCHYCGM |
| 49 | 1400.6 | 1578.556 | 3 | 4732.62 | 0.0257 | 5.4 | DNDQKTGWMMMRHCWWWWYYYYQMDDCMCCDDCEE |
| 50 | 1448.7 | 1570.071 | 3 | 4707.128 | 0.0633 | 13.4 | DAKTRRRRRRRVWWWWWWWWWWYGGFCCDMDDD |
| 51 | 1358.5 | 1551.648 | 3 | 4651.988 | -0.0667 | -14.3 | DMMTYMMCCCDCCCYVVRGPRRWKCRRRRHKKVDWD |
| 52 | 1406.3 | 1540.672 | 3 | 4618.949 | 0.044 | 9.5 | DACNEMMMMNYDDGYEDDCEGEWRQLKARRLHVYKRT |
| 53 | 1349 | 1534.882 | 3 | 4601.561 | 0.0614 | 13.3 | QRRRRRRRRAAHEGGCYRRRYSSLRFFRRRRMLVV |
| 54 | 1331.3 | 1524.511 | 3 | 4570.458 | 0.0529 | 11.6 | DDFEKRRKKRLFCPWRMMFAQFVKKKKKKKTTFMAG |
| 55 | 224.6 | 1521.977 | 3 | 4562.976 | -0.0682 | -14.9 | DAWWMPGNQMMGDDMDNWWWMMMKKKLRRLNYFR |
| 56 | 218.6 | 1521.966 | 3 | 4562.943 | -0.066 | -14.4 | DAWWWMMRRQMDCDMMMMMSSSCRRSRERRHKKL |
| 57 | 990.2 | 1510.377 | 3 | 4528.16 | -0.0508 | -11.2 | DAWWWHHFGGHCGRRRRKKKRPLPCPWWWFYDDY |
| 58 | 1035.7 | 1507.844 | 3 | 4520.547 | -0.0359 | -7.9 | DQCCCYYMMMMCMEDDCCCCNCCCCGERRRRRRVPGL |
| 59 | 1378.8 | 1495.685 | 3 | 4483.968 | 0.0649 | 14.5 | DAKKFRRRYRRMMMMMMMCHGEWWWMGRGKWGF |
| 60 | 599.5 | 1491.934 | 3 | 4472.828 | -0.0461 | -10.3 | LLRLMEEEMMPTMYYMDKWHENEESGEEGWRNENS |
| 61 | 1092.6 | 1491.698 | 3 | 4472.106 | -0.0343 | -7.7 | DDAWWMMCSTDFVSMMLRRRRRRSPKWWRRYML |
| 62 | 1087.7 | 1472.414 | 3 | 4414.36 | -0.1389 | -31.4 | MMMCCCCDMMCDGNMDGEWWWWDRMGEMHNYCWN |
| 63 | 1459.4 | 1467.88 | 3 | 4400.67 | -0.0536 | -12.2 | LLPLLPWLVHRKWRMEGMMCCCCCDCGNDMDCCMDDD |
| 64 | 1254.6 | 1462.685 | 3 | 4385.07 | -0.0359 | -8.2 | DPPDYMMMMEERFKLYRRRRRRRRFYFGGMCR |
| 65 | 1335.5 | 1460.873 | 3 | 4379.653 | -0.054 | -12.3 | DRHWMDMMERFRHLGEWWYMMQMEGMEDDMMQ |
| 66 | 1212.1 | 1452.308 | 3 | 4353.957 | -0.0534 | -12.3 | DADNNRMMMKGEWWYYMNWWMRKKLALLVYSSSS |
| 67 | 599.3 | 1450.536 | 3 | 4348.603 | -0.0154 | -3.5 | DAWWVKTRRRRRLKKLLKKRRRLYKAWVSHCKK |
| 68 | 1355.2 | 1437.777 | 3 | 4310.322 | -0.0141 | -3.3 | LLAKLLEMERFKNWWDAEYKKRPPWKKKKLMSST |
| 69 | 1305.7 | 1431.785 | 3 | 4292.359 | -0.0262 | -6.1 | QCCCCCCCCCRESVWMMMMMMMMMEEGHRKQMMQ |
| 70 | 1463.7 | 1429.598 | 3 | 4285.821 | -0.0501 | -11.7 | YECDWMHWMDWKKAVVKLLLLHEGMMHVCDMDDD |
| 71 | 1218.6 | 1421.978 | 3 | 4262.975 | -0.062 | -14.5 | DPNNHHSWMMMMCYDWVSRRRRRVPLCWRRKP |
| 72 | 723.1 | 1421.982 | 3 | 4262.953 | -0.0289 | -6.8 | DAVSWWYQKHKRRDPLVLLWWWYFQENNCCMM |
| 73 | 685.9 | 1421.974 | 3 | 4262.945 | -0.0454 | -10.6 | DWWWQWWWWWMMMCMMQCGRRRLKPRRKR |
| 74 | 624.3 | 1421.975 | 3 | 4262.893 | 0.011 | 2.6 | DAWWFRRYMWWMAYMMRNQLPMWWWYVGER |
| 75 | 1220.9 | 1421.976 | 3 | 4262.889 | 0.0164 | 3.8 | RLVVKKKRTLKAEWYRRRDCSCCDMMDDCCDMMQ |
| 76 | 515.4 | 1421.306 | 3 | 4260.874 | 0.0224 | 5.3 | DADAWWWMMTPYGRARRQLHHRNWWWWWCEE |
| 77 | 203 | 1421.304 | 3 | 4260.828 | 0.0629 | 14.8 | DAHQSRVGPSQFMMMMMMMWYECWWTRRRKPR |
| 78 | 540 | 1421.307 | 3 | 4260.762 | 0.1377 | 32.3 | VVKLLLLLLRVSWERRARKVVRRVVKKKKLYFLR |
| 79 | 1427.2 | 1419.371 | 3 | 4255.083 | 0.0091 | 2.1 | DHNYEGVSLCGADGLLLVVLKKKWRFTNETASHTYMSG |
| 80 | 1427.9 | 1416.25 | 3 | 4245.784 | -0.0553 | -13 | DDNQKQTAPGCSTAPADGHASVYMTADPYLEETHATMGYS |
| 81 | 1149 | 1414.812 | 3 | 4241.429 | -0.0154 | -3.6 | HGVMTSWKKAGKRRLLLLRRRRAKGKLLTFQVMMQ |
| 82 | 1134.9 | 1408.144 | 3 | 4221.465 | -0.0543 | -12.9 | MRHWCMMDFWYMHRRGRGEYDCCCCCCCPAMQM |
| 83 | 1130.7 | 1394.963 | 3 | 4181.903 | -0.0368 | -8.8 | DWWRRRFKYMMMMMFFMMCYVSRRRRRVM |
| 84 | 1079.2 | 1391.715 | 3 | 4172.063 | 0.0593 | 14.2 | KLLHRRRLWWWWWWWWWWEGSPKMQVSYN |
| 85 | 1338.2 | 1386.198 | 3 | 4155.511 | 0.0612 | 14.7 | YLWGFRRLLRRRRRRRRRFKKHKKVLLPGDH |
| 86 | 1294.9 | 1383.582 | 3 | 4147.688 | 0.0348 | 8.4 | LLLLELKLLWMPNGGPKHMMCCCCCCWMMMMMMQ |
| 87 | 1083.3 | 1375.17 | 3 | 4122.436 | 0.0526 | 12.8 | LLLRLPVGHLLWWYMNRRRRRRRRRRRVPW |
| 88 | 1441.1 | 1371.195 | 3 | 4110.614 | -0.05 | -12.2 | DMCCCCCCMYMDDDFHRKKRRREFMFRLYCGM |
| 89 | 1033.6 | 1369.69 | 3 | 4105.995 | 0.0542 | 13.2 | KPQWRSPKLLLRRRMRKKKWEDDCCCCCCCHVV |
| 90 | 1298.9 | 1367.957 | 3 | 4100.885 | -0.0362 | -8.8 | DHVGQAWWYMMHPDGWWWDDCRKKKKVHLMLA |
| 91 | 1338 | 1366.612 | 3 | 4096.852 | -0.0364 | -8.9 | DAKVAFRLPWWVSFLRNMMDCMMCCDPVVVLMMQ |
| 92 | 1080.8 | 1364.994 | 3 | 4091.93 | 0.0314 | 7.7 | LLLLLKLPWFKLKPKLLMMCLMMMCDMMMCGMM |
| 93 | 1434.4 | 1362.462 | 3 | 4084.36 | 0.0052 | 1.3 | KRPRRRRKNNKCWDYNVVKLLWYRLVSRLLR |
| 94 | 351.9 | 1349.151 | 3 | 4044.474 | -0.0412 | -10.2 | MMGHNKPGMMCCCMWWWWWWYMDYRTLDDC |
| 95 | 1198.1 | 1348.389 | 3 | 4042.091 | 0.053 | 13.1 | KLAKGPKLVRRYERARRRRRKKKMMMHCCCCC |
| 96 | 926.9 | 1346.464 | 3 | 4036.319 | 0.0508 | 12.6 | RLWRTPKWRMRKRRLKARRRFWWWRPQK |
| 97 | 1307.2 | 1346.012 | 3 | 4035.072 | -0.0586 | -14.5 | DRHWWMMQRRHRRERRRERRRRRVGESSS |
| 98 | 1436.2 | 1345.433 | 3 | 4033.232 | 0.0457 | 11.3 | YLFEGAWWYMYYYRRRKKKFVKLATTKLLVL |
| 99 | 1401.8 | 1335.866 | 3 | 4004.636 | -0.0583 | -14.5 | DDDMHCDDDCPGYYNWFKRKKKLTKENNGMGCM |
| 100 | 1341.8 | 1996.992 | 2 | 3991.921 | 0.0486 | 12.2 | RLLNLVALWWWWWWWYRKYTWWSSDYEN |
| 101 | 1380.2 | 1330.452 | 3 | 3988.278 | 0.0563 | 14.1 | YLMMMDREEGMMWDNCRRCSGCCCCCCCCPFNE |
| 102 | 1322.3 | 1330.377 | 3 | 3988.107 | 0.0025 | 0.6 | DHDGMMCCQNYMMMMMMDMCNFGLCCCCCGMMQ |
| 103 | 1393.1 | 1992.782 | 2 | 3983.491 | 0.0584 | 14.6 | KLARKRMGAKLVVFMCGCMCCCCMDCCMDDDFGCM |
| 104 | 1345.5 | 1992.375 | 2 | 3982.744 | -0.0093 | -2.3 | DPPDWVPPKGEFNWWKKWKYMMQQYMNMMQ |
| 105 | 1029 | 1323.986 | 3 | 3968.983 | -0.0469 | -11.8 | DAWWWWMAMFMWRHRRRRRDWWRHKKL |
| 106 | 906.5 | 1321.985 | 3 | 3962.991 | -0.0586 | -14.8 | DSSWMMCCEWWWWWRWHKRLLLKKLLLVL |
| 107 | 1533.4 | 1321.986 | 3 | 3962.988 | -0.0524 | -13.2 | DEQERRAKRSGWMMMMMMMNRKKLLLLLLVL |
| 108 | 970.8 | 1321.981 | 3 | 3962.98 | -0.0577 | -14.5 | DAMMGWAAFWWWWWVCMHDNRVLKKLLLLLL |
| 109 | 948.3 | 1321.984 | 3 | 3962.946 | -0.0152 | -3.8 | DAKKLKARTYWESWMDSHHSVRKKLFMWDNPG |
| 110 | 1238.6 | 1321.986 | 3 | 3962.893 | 0.0424 | 10.7 | NCSHWMMRHWKQLLRRRRKLEGRMMHHGND |
| 111 | 1224.3 | 1321.982 | 3 | 3962.885 | 0.0392 | 9.9 | DSSHFLVLLQVWWWWGERLRRVYEYMSQMM |
| 112 | 823.9 | 1321.983 | 3 | 3962.871 | 0.0559 | 14.1 | KRKKVLLLRQRHMMQNMAHGCGEWWWWMEM |
| 113 | 848.9 | 1321.98 | 3 | 3962.868 | 0.0515 | 13 | LKAHKKLRRRRLWWMWHDWWMMEENMDM |
| 114 | 995.2 | 1321.661 | 3 | 3961.906 | 0.0559 | 14.1 | HEGVRREWWWFNRRRRWLWVKDWMMEEA |
| 115 | 516.1 | 1321.316 | 3 | 3960.968 | -0.042 | -10.6 | DEGAMMMCRRLLVKRRALKKKKKLLEGEDDMCC |
| 116 | 404.4 | 1321.319 | 3 | 3960.966 | -0.0326 | -8.2 | RLRQWAAKFCCRWWWWWYYRYELARKPPG |
| 117 | 596.2 | 1321.311 | 3 | 3960.863 | 0.05 | 12.6 | DHAWWWKWWWWWRRRRRFLWEGWPGCM |
| 118 | 1389.3 | 1977.788 | 2 | 3953.515 | 0.0468 | 11.8 | QLRSHWVSWWWKWWFFYMEMCCDCCMDDD |
| 119 | 1136.1 | 1317.134 | 3 | 3948.357 | 0.0228 | 5.8 | DNNPLAPKWYGEMAYMEDYECCCEMDDEDMMQ |
| 120 | 839.8 | 1316.256 | 3 | 3945.798 | -0.051 | -12.9 | DAWSGMAKWWKYSWWWWWRKWWWWWW |
| 121 | 1337.5 | 1312.715 | 3 | 3935.146 | -0.0226 | -5.7 | DARVMYMFLLGRLLLFKKKKKKKSGGMNGGVYEN |
| 122 | 1460.2 | 1966.185 | 2 | 3930.297 | 0.0581 | 14.8 | DMECWGNEWRGVRHCMVMMMMMQMDCCMDDD |
| 123 | 1346.7 | 1965.034 | 2 | 3928.03 | 0.0232 | 5.9 | DLRRRMKKKKGHDWHCCMCCGKKKKWRLPRT |
| 124 | 1342 | 1301.042 | 3 | 3900.08 | 0.0232 | 6 | DCCDMCMDMMGHNGYYEMVGGGHCCPCCCCCGMMQ |
| 125 | 1348.6 | 1297.096 | 3 | 3888.218 | 0.0494 | 12.7 | LLCRRKHYKVWWWRRRWMSLYVRKKLLL |
| 126 | 1320 | 1943.158 | 2 | 3884.3 | 0.0029 | 0.7 | DAHTYECNRKKKLLLKKKKKKKKKKRPLVGCM |
| 127 | 1373.6 | 1926.416 | 2 | 3850.781 | 0.0376 | 9.8 | LLKNRFQYMMCCNCMMCMHKKKLVKKLMCR |
| 128 | 1177.9 | 1925.504 | 2 | 3849.044 | -0.0513 | -13.3 | SQCCCCCNCCWWWNSTDWCCCDGGMMCMDMMQ |
| 129 | 1328.2 | 1913.502 | 2 | 3825.046 | -0.0563 | -14.7 | DDDDCNCCCCCCMMMWYMMMRMMYSSSMMQ |
| 130 | 1312.7 | 1912.31 | 2 | 3822.596 | 0.0102 | 2.7 | DSSAFRHAWYQWWMSWWWYMQYSRGRCM |
| 131 | 1439.4 | 1273.248 | 3 | 3816.667 | 0.0539 | 14.1 | DAGVVFEPPKDCDEFKKKKKKKCMCCEESMDCS |
| 132 | 1258 | 1901.808 | 2 | 3801.625 | -0.0248 | -6.5 | DPPDYDWWWWLLKKLLLQMMMMMMDMGMA |
| 133 | 1409.4 | 1887.143 | 2 | 3772.313 | -0.0409 | -10.8 | DDNQMHCCWWRNQCCNDGFVAMHMMPDGCYM |
| 134 | 1490.1 | 1879.06 | 2 | 3756.163 | -0.0563 | -15 | DFMTSMMCWYYWMEDDDGENPDMMCCMDDD |
| 135 | 1377.1 | 1877.177 | 2 | 3752.306 | 0.0331 | 8.8 | DVHGHQKMVCDCCDDDSMDMDACDMCHKKMMQ |
| 136 | 1478.1 | 1250.671 | 3 | 3748.941 | 0.0493 | 13.1 | RLVPLAWRRTKKKKKKKKLLMMCCCCCMQM |
| 137 | 1457.5 | 1857.732 | 2 | 3713.395 | 0.0546 | 14.7 | DYSECCDMWWADFRRRRRESSFDCCMDDD |
| 138 | 1207.8 | 1855.233 | 2 | 3708.398 | 0.0532 | 14.3 | DNNPRVGFKRAAAAFKCMMMHECCCCCPCMCTA |
| 139 | 666.6 | 1236.723 | 3 | 3707.095 | 0.0519 | 14 | KRPPKWHKRWWRWRRWRRRRRYRY |
| 140 | 1415.8 | 1222 | 3 | 3663.03 | -0.0527 | -14.4 | DECCNWMDPMWYEMCCMCCCPCMQSSYECG |
| 141 | 1175.7 | 1221.992 | 3 | 3663.008 | -0.0531 | -14.5 | YLRMYYMWWRMQKKKKKRRRGARRQK |
| 142 | 1549.9 | 1221.991 | 3 | 3663.005 | -0.0535 | -14.6 | DCAECAMMDMQTDDCCCCDDCCCWWWMPHM |
| 143 | 1319.6 | 1221.991 | 3 | 3663.001 | -0.0488 | -13.3 | DDCCQKRRLRRTRRRRRRRRRRFYNE |
| 144 | 997.9 | 1221.989 | 3 | 3662.974 | -0.0273 | -7.4 | DCGEADCAMEWMMCDCDDMMMESSDMMMMCC |
| 145 | 948 | 1221.987 | 3 | 3662.964 | -0.0247 | -6.7 | DDCDDYYMMMCCSFERMMCCCCCCCDATDGM |
| 146 | 315.4 | 1221.99 | 3 | 3662.926 | 0.0212 | 5.8 | YLDKMMSRKKKKKKLLGVEYGRHRMMHHA |
| 147 | 400.8 | 1221.991 | 3 | 3662.907 | 0.044 | 12 | YLTRKKLLKLWWWWWWWWRMQEVQQ |
| 148 | 1485.9 | 1221.989 | 3 | 3662.894 | 0.0501 | 13.7 | FLFKKVKLLLLFLYYRRRRFMDCCMEPC |
| 149 | 272.3 | 1221.989 | 3 | 3662.892 | 0.0533 | 14.5 | DAWWRRKRLCYFWDYLRSYRRRRREA |
| 150 | 839.5 | 1221.989 | 3 | 3662.891 | 0.0545 | 14.9 | MLAWWQRQYYRKRKRREKWFEGKRMM |
| 151 | 861.6 | 1221.718 | 3 | 3662.136 | -0.0053 | -1.5 | DDDCMDMENCYQLWNMWWMMMDECYCY |
| 152 | 1068.1 | 1221.656 | 3 | 3661.953 | -0.0073 | -2 | DAMMMDEGEMCCDCDMMMMSCMWEMHCCSD |
| 153 | 90.1 | 1218.672 | 3 | 3653.019 | -0.0255 | -7 | DEAHTYYTHEKSRRRRRPMLNRRKKKKL |
| 154 | 1388.2 | 1825.707 | 2 | 3649.352 | 0.0471 | 12.9 | SKLLKKMHCCNMCCGCACMMLPPLMMCCMDDD |
| 155 | 1438.5 | 1825.659 | 2 | 3649.262 | 0.0417 | 11.4 | LAPAMQRRYRMKVMCCCCCCWMCCCDMDDD |
| 156 | 1395.4 | 1816.11 | 2 | 3630.241 | -0.0359 | -9.9 | DVSTEMMDEWEWWWRMYMQGNCCDSDCM |
| 157 | 1346.3 | 1207.094 | 3 | 3618.214 | 0.0455 | 12.6 | LLGSAPKRKKKRLQARRRRLGEKRKAKFEN |
| 158 | 1213.5 | 1206.588 | 3 | 3616.723 | 0.0204 | 5.6 | DNPDKKKERVTEWWYMDCGVRRAALLMSST |
| 159 | 649.1 | 1809.059 | 2 | 3616.074 | 0.0295 | 8.2 | DVSYTVRKKKLLLRRRRRRRRECSMGNLA |
| 160 | 766 | 1807.782 | 2 | 3613.496 | 0.0521 | 14.4 | DCMWWWWWYMNCHSWCSYQGKKKEVDL |
| 161 | 1136.9 | 1806.573 | 2 | 3611.164 | -0.033 | -9.1 | QCCCCCCCCCCCCCMDELWLRHKLYYMGYQ |
| 162 | 1286 | 1204.545 | 3 | 3610.667 | -0.0537 | -14.9 | ELMHPWWWWDWYMYMKLLLLLHDRCM |
| 163 | 1333.3 | 1201.754 | 3 | 3602.223 | 0.0178 | 4.9 | LLYLRAPFKTFKKKKRRKVRKKHKVFQM |
| 164 | 1320.6 | 1196.99 | 3 | 3587.981 | -0.0328 | -9.1 | DAHSPEMMCCCCNMMDDDDCDNMMDPDMQM |
| 165 | 103.5 | 1195.765 | 3 | 3584.222 | 0.0499 | 13.9 | DQLKWWRRHADYMMMMMMHCCCCNCCGC |
| 166 | 1284.1 | 1790.379 | 2 | 3578.708 | 0.0357 | 10 | DRHAFKLAQGVKLWWWWWWWMCVARCM |
| 167 | 1293.9 | 1190.242 | 3 | 3567.658 | 0.0453 | 12.7 | DTRPRPRYFWWMYMECERRRRRMQM |
| 168 | 1084 | 1188.86 | 3 | 3563.504 | 0.0531 | 14.9 | DKVKKKWMLMMMMMMMMMMYSSLLFHA |
| 169 | 1383.2 | 1778.195 | 2 | 3554.348 | 0.0277 | 7.8 | KVFWYMYNMMMMDDMMMYRPPPEFGDA |
| 170 | 82.2 | 1185.737 | 3 | 3554.24 | -0.0512 | -14.4 | DAWWWMYREWYTTTREMCCSDCCECCY |
| 171 | 398.4 | 1182.081 | 3 | 3543.256 | -0.0332 | -9.3 | DVPKKVLRFFKLRRRRRRHKKRRVGKK |
| 172 | 1220.7 | 1167.856 | 3 | 3500.591 | -0.0467 | -13.3 | DAGYGSWWWWYWWEEFVWYFKWPLK |
| 173 | 1316.7 | 1164.837 | 3 | 3491.537 | -0.0475 | -13.6 | MLMMSCNPWWWWWYQQRVRVHFRCM |
| 174 | 1082.4 | 1164.74 | 3 | 3491.16 | 0.0392 | 11.2 | KLSVLYRWRRRRRRRRRRRRRELR |
| 175 | 856.7 | 1158.65 | 3 | 3472.895 | 0.0339 | 9.8 | FLLKKKKLVRKKKKVMQYYYMDRMEM |
| 176 | 1088 | 1735.831 | 2 | 3469.698 | -0.0508 | -14.6 | DWMDCMCGCCCYMDDDCMYCCCCCCCCACC |
| 177 | 1229.1 | 1735.73 | 2 | 3469.492 | -0.0457 | -13.2 | DYYMDWMHGKKKKKLRMYMMEECCFQ |
| 178 | 1417.7 | 1156.86 | 3 | 3467.6 | -0.0423 | -12.2 | DRVKMLGMWWKLWRRRYMKDDDMMCA |
| 179 | 1324.1 | 1733.033 | 2 | 3464.017 | 0.0335 | 9.7 | SYNCCCCCCCAAWWWWHQMMCCCCPMMQ |
| 180 | 1440.7 | 1730.558 | 2 | 3459.143 | -0.0422 | -12.2 | DMMTWYCKKLLVMMMMMGGCCCGCEECCD |
| 181 | 1361.4 | 1730.487 | 2 | 3459.001 | -0.0405 | -11.7 | DVMFWWWRHKKLKKVLGERKKLYKKL |
| 182 | 1347.6 | 1727.707 | 2 | 3453.449 | -0.0503 | -14.6 | DMCACCDCCMDMCNHRAPAYERRKKKVLL |
| 183 | 1334.4 | 1150.531 | 3 | 3448.603 | -0.0317 | -9.2 | LLRDYDDWTNYMMMEYKEEGPGKLLPPL |
| 184 | 1307 | 1723.881 | 2 | 3445.73 | 0.0172 | 5 | SVPPDYYWWWWSAWFKKKRTLLLMMQ |
| 185 | 1343.8 | 1720.669 | 2 | 3439.375 | -0.0514 | -14.9 | LLLTMTTRSWWWQLMMMMMCCPEMMQ |
| 186 | 1022.5 | 1147.251 | 3 | 3438.683 | 0.0482 | 14 | WLKRARRRRLTLKLLLYMMECMMCCCC |
| 187 | 1228.2 | 1719.315 | 2 | 3436.624 | -0.0075 | -2.2 | SCPHYMNSVRRLMEKLWRRLYFNSLCC |
| 188 | 1262.7 | 1709.558 | 2 | 3417.095 | 0.0077 | 2.3 | LLLVVKKKLFKKVWGEKKKRFFGEWGLL |
| 189 | 1302.8 | 1708.826 | 2 | 3415.619 | 0.0191 | 5.6 | MLVWWWWWWWWGEEGRRRRYLYN |
| 190 | 1249.3 | 1138.617 | 3 | 3412.781 | 0.0486 | 14.2 | DASYMKKKKGWWWYYRKVGKFFQQRV |
| 191 | 1452 | 1706.572 | 2 | 3411.085 | 0.0457 | 13.4 | DCMEMCSMFMLGEWREMMQMMCSMDDD |
| 192 | 1317 | 1136.886 | 3 | 3407.682 | -0.0454 | -13.3 | DAERWWMMRRLLWGEEGRRRKHYKM |
| 193 | 1329.5 | 1134.308 | 3 | 3399.921 | -0.0195 | -5.7 | DWMWLTGVKKLTLLVVPLTGRVLVKLVGMC |
| 194 | 1337.8 | 1697.908 | 2 | 3393.802 | -0.0004 | -0.1 | WLHTYRYVVGKVVPKLVPHKNETGGLQME |
| 195 | 1156.3 | 1129.51 | 3 | 3385.467 | 0.0427 | 12.6 | DMGHCCHVFFRWWKWWWRKRNMCGD |
| 196 | 1423.4 | 1128.674 | 3 | 3383.039 | -0.0387 | -11.4 | DTTNWEGPMTYFMHCCCCHEMDDDSDCM |
| 197 | 1432.1 | 1692.129 | 2 | 3382.199 | 0.0447 | 13.2 | DARVHRKLHNWMCCCCWMMMDCCMDDD |
| 198 | 133 | 1121.999 | 3 | 3363.016 | -0.0417 | -12.4 | DDDDDMHTMDCCDDEWWRMELMECCCC |
| 199 | 180.3 | 1121.998 | 3 | 3362.935 | 0.0366 | 10.9 | LLLLLLLRHQWRRKKSVEGRFNRMGVE |
| 200 | 250.5 | 1121.992 | 3 | 3362.903 | 0.0496 | 14.7 | DVKKVREWWADWWRFKKKKKKKVNF |
| 201 | 1430 | 1679.737 | 2 | 3357.419 | 0.0398 | 11.9 | SLGRRRRRGRPAWHWMCCGGMEDDMMCG |
| 202 | 108.5 | 1117.748 | 3 | 3350.173 | 0.0494 | 14.7 | DAWMHKMCCCYMMMGGCCWCKQHSSGNR |
| 203 | 1138.4 | 1673.769 | 2 | 3345.48 | 0.0432 | 12.9 | DPDLWWRRRGGPTKKQWEGMMMMMCTA |
| 204 | 1370 | 1113.728 | 3 | 3338.124 | 0.0381 | 11.4 | DARKLKKKSGFKKKKKKVVEGSRRLLLGL |
| 205 | 1056.7 | 1661.216 | 2 | 3320.452 | -0.0356 | -10.7 | QLKWWWWWWWWWWWTHPEMCTA |
| 206 | 1298 | 1658.373 | 2 | 3314.688 | 0.0446 | 13.4 | DVGHWWWHRPGASQRRRRRRRGGRMC |
| 207 | 1283.7 | 1105.231 | 3 | 3312.672 | -0.0014 | -0.4 | DPGVKPKKKLFLYRRAGEFEHYMNMMQ |
| 208 | 1347.9 | 1640.281 | 2 | 3278.569 | -0.0206 | -6.3 | DVLWQAARWWWMHGERKSKKKDMGMA |
| 209 | 1231.8 | 1638.522 | 2 | 3275.069 | -0.0388 | -11.8 | SCAYYYMMMMMMMMMEYMDGVVMGMA |
| 210 | 1061.2 | 1091.65 | 3 | 3271.881 | 0.0481 | 14.7 | DWGERLRKPWRRRRRKGLLWWRPL |
| 211 | 1250.5 | 1090.148 | 3 | 3267.418 | 0.0042 | 1.3 | GGYMYWRGSPDSVMHLLDPSWAHPAYEN |
| 212 | 1323.9 | 1627.676 | 2 | 3253.294 | 0.0422 | 13 | ACKYFDRRMRRKWMMHMECCCMAMG |
| 213 | 1017.2 | 1623.229 | 2 | 3244.491 | -0.0471 | -14.5 | DAYMPEWWYDMCLLALHLSRRRHHN |
| 214 | 1337.3 | 1620.843 | 2 | 3239.631 | 0.0413 | 12.7 | DAHNMRREWYMEKKKKLLCAKVGGYW |
| 215 | 1343.5 | 1612.107 | 2 | 3222.222 | -0.0213 | -6.6 | DYDEWFKKKKFPHMCCDMCDECMQM |
| 216 | 1082.6 | 1610.209 | 2 | 3218.451 | -0.0482 | -15 | DAWWWWVSMWEESDWWRRRRFF |
| 217 | 1322.1 | 1608.429 | 2 | 3214.798 | 0.0452 | 14 | DRHVLLLLKKAGAGLVRFEEYRLLGGYE |
| 218 | 915.4 | 1608.38 | 2 | 3214.77 | -0.0251 | -7.8 | FAAWGEWRKRRRKKKVWWWWRRG |
| 219 | 1021.7 | 1608.376 | 2 | 3214.691 | 0.0465 | 14.5 | WLQRRRAWWWWWWWRRRVGRF |
| 220 | 289.6 | 1608.359 | 2 | 3214.69 | 0.0132 | 4.1 | MWWMMMMMYPYRKKKKKKKKLKL |
| 221 | 1315 | 1597.127 | 2 | 3192.285 | -0.0471 | -14.7 | HVGDCCCGCGGCHPPHHHEKSYRRQFEN |
| 222 | 1463 | 1061.572 | 3 | 3181.729 | -0.0346 | -10.9 | DTRRFKKKWWWYRRRRRKSSHY |
| 223 | 1367.1 | 1061.146 | 3 | 3180.384 | 0.0312 | 9.8 | LHSAMMCNMDPEPTGYYMLLALLLLCSC |
| 224 | 1370.9 | 1590.726 | 2 | 3179.41 | 0.0274 | 8.6 | DAHTYFNHHRRRAGRREGFEMDMMQ |
| 225 | 1364.5 | 1582.669 | 2 | 3163.324 | -0.0009 | -0.3 | DVGHWWWWRPGGEERRLNCCCPMMQ |
| 226 | 92.4 | 1568.632 | 2 | 3135.279 | -0.0293 | -9.4 | DAGGGMYWWWWWWWRRAMMEGDSN |
| 227 | 1080.6 | 1566.348 | 2 | 3130.704 | -0.022 | -7 | HRRVQWWFLRTWWKLYYRLAGPL |
| 228 | 1429.2 | 1042.913 | 3 | 3125.753 | -0.0362 | -11.6 | DAECCNDDCMQMNCCCCCMGEECMCACM |
| 229 | 1262.4 | 1560.231 | 2 | 3118.4 | 0.0461 | 14.8 | SPPKQLLRHGGTPQLWYGCCGHMMRCM |
| 230 | 361.4 | 1558.558 | 2 | 3115.116 | -0.0148 | -4.7 | SVTKKCDGCCYMHCCCYECCCCSWLLL |
| 231 | 1329.9 | 1557.299 | 2 | 3112.556 | 0.0281 | 9 | GYGMMHEDYRERHKKKKKKLLMMGA |
| 232 | 87.5 | 1556.609 | 2 | 3111.161 | 0.0421 | 13.5 | LLLLLLLRVLALVLKKVVGRVRFKLLL |
| 233 | 1350.2 | 1037.272 | 3 | 3108.76 | 0.0344 | 11.1 | KQLTTHTLLGVKREYKHKFKTYKVY |
| 234 | 1340.2 | 1553.3 | 2 | 3104.624 | -0.0376 | -12.1 | DHPPAWYAGYQLPGEQWKKLRELPLL |
| 235 | 679.2 | 1033.768 | 3 | 3098.299 | -0.0172 | -5.5 | DAYYWVDKMEDDSRFKWEGEEDRN |
| 236 | 1443.9 | 1550.108 | 2 | 3098.245 | -0.0441 | -14.2 | DMETRRRWKRKLMCDDMMCEECCD |
| 237 | 112.7 | 1031.7 | 3 | 3092.112 | -0.0349 | -11.3 | DAMCMCCTCCCYWFFCYSSYMKKRT |
| 238 | 1438 | 1026.951 | 3 | 3077.875 | -0.0455 | -14.8 | DLEDMDDCHHCMCMEEDDEDMGFCM |
| 239 | 1343.3 | 1538.604 | 2 | 3075.15 | 0.044 | 14.3 | DAWRLLHCQWTKMMGMMMCCDMMQ |
| 240 | 1375 | 1523.244 | 2 | 3044.428 | 0.0451 | 14.8 | DHRMAHWWMHCKKLLEFELLMDK |
| 241 | 173.2 | 1521.964 | 2 | 3041.941 | -0.0265 | -8.7 | SPPGFKKKLKVVVLRRRVPKERRRL |
| 242 | 321.5 | 1521.97 | 2 | 3041.938 | -0.0121 | -4 | KLAFFKFKVRKKYRKLLLYRKRL |
| 243 | 179.1 | 1521.978 | 2 | 3041.916 | 0.025 | 8.2 | DARKRTRRLLRVRRKLYYKLLRL |
| 244 | 1172.2 | 1514.468 | 2 | 3026.947 | -0.0252 | -8.3 | DMGHWWWMMMMMYMCYCSSMQM |
| 245 | 1206.5 | 1512.322 | 2 | 3022.637 | -0.008 | -2.6 | AYRGGKLAKKLLPKYYYRVGAWMYQ |
| 246 | 1444.9 | 1501.118 | 2 | 3000.186 | 0.0366 | 12.2 | DYDTMDCCWGPEMEPQYRRLSMDK |

**Table S2.** Analysis of peptides in fraction 5 using novor.cloud

| No | mz(data) | z | pepMass  (denovo) | Err  (data-denovo) | ppm | Peptide |
| --- | --- | --- | --- | --- | --- | --- |
| 1 | 1618.988 | 4 | 6471.897 | 0.0274 | 4.2 | DMSDSQWWYWECLCPDCCCCREWFSWHERRRRKKRRHRNKLVVCPGQ |
| 2 | 1987.846 | 3 | 5960.588 | -0.0701 | -11.8 | QCMMGYCWMMDVRRRRRRRGAHTYRFKWRRKDVMMDGMMMGMDDD |
| 3 | 1985.624 | 3 | 5953.982 | -0.1327 | -22.3 | SMMMCCCCNWWWMCCCCCCAFLQRMCCCCCNYYWERRSMR |
| 4 | 1981.168 | 3 | 5940.565 | -0.0834 | -14 | DHNQWWCCCCTWCECCCRMTHLEECYRRFFPKKLVKKPPKMTGT |
| 5 | 1934.35 | 3 | 5800.099 | -0.0711 | -12.3 | DMMTCHDDMMMEDDDWCCCMDDNGYLKAKKWYRRSPKMGMDMDDD |
| 6 | 1879.86 | 3 | 5636.576 | -0.0177 | -3.1 | DRHWFLRRAWYRRKFSVWKNYYCMCMDDWCECVGKRPLMKD |
| 7 | 1864.458 | 3 | 5590.431 | -0.0775 | -13.9 | DAHSMMMDCCCCDDDECMPSCCCCCCPDQCCCCQCCSSCMM |
| 8 | 1864.223 | 3 | 5589.727 | -0.079 | -14.1 | DHHCCCCCCEAWWYYAGMDWKWYCCCCCCCCCYFGGCVM |
| 9 | 1861.425 | 3 | 5581.321 | -0.067 | -12 | DKECCCCCCCDRRRTHPAWWWYYRRRKRECRHRMHCMM |
| 10 | 1857.873 | 3 | 5570.671 | -0.0724 | -13 | MYCCMGCCMMYCCECYSVYVMKKCCCCMDGCMHDDMDYCM |
| 11 | 1835.749 | 3 | 5504.169 | 0.0547 | 9.9 | MGVCCCCRMHWNWWSMDWWWWWWWWWWRRRFCEGDGS |
| 12 | 1821.628 | 3 | 5461.931 | -0.0699 | -12.8 | MMMTWWRRRKKLTWKVLYKLLSFAFPKVGVRRRRRRCCCVM |
| 13 | 1807.625 | 3 | 5419.921 | -0.0665 | -12.3 | DACAACCDMCESCHHMMMMCSCDEMWCYRYRRRKKQYMC |
| 14 | 1800.669 | 3 | 5399.014 | -0.03 | -5.5 | DAWWYFQCCCPDCCYKWWWKLCCCCCDNCGGWVSYRRM |
| 15 | 1800.142 | 3 | 5397.422 | -0.0188 | -3.5 | FNMYNYWCLCWPPRPPVWWRFKWWWYYCCCRRLVMMK |
| 16 | 1780.277 | 3 | 5337.874 | -0.0662 | -12.4 | DMMTYCEMMMGCSSMKKGRQYRRTWHCHSSYCCCMCMDDD |
| 17 | 1773.22 | 3 | 5316.704 | -0.0656 | -12.3 | DEVPWRKKKRRRHWEYDCWWWWMKYKLVKRRFKLMQM |
| 18 | 1771.403 | 3 | 5311.134 | 0.0525 | 9.9 | DRPPVRRRKRRVVVDDDPHKKKKSSTKLYRKKLRRRVLRMTGT |
| 19 | 1770.272 | 3 | 5307.823 | -0.0293 | -5.5 | DACCCHHCCYCDMWYYCRWSVWWRLYCMMCCCVCVM |
| 20 | 1747.118 | 3 | 5238.4 | -0.0675 | -12.9 | DRHRKLLHKKKLPPCHETAGDDHEVGWRRRTMMDGMEDDCMY |
| 21 | 1738.474 | 3 | 5212.467 | -0.0679 | -13 | DQNMMMMMCCCCCECCCCCMSWFFYYRCMMMDMDDD |
| 22 | 1723.044 | 3 | 5166.036 | 0.0742 | 14.4 | KLRHPKKWWWMEDDNYYWECRTWWWCCCCCCDFNE |
| 23 | 1708.602 | 3 | 5122.826 | -0.0422 | -8.2 | MRHYYNWCWYCCYLTCCCPTWWWEGGECECQSHMQM |
| 24 | 1688.96 | 3 | 5063.915 | -0.0575 | -11.4 | DWWRRDWWCCGWDWYWYRRMRWCCCCCWYCSSN |
| 25 | 1687.543 | 3 | 5059.572 | 0.0341 | 6.7 | DVGHDARRRHKKKLRRVWWCRRMWWWWEGRRRHYEN |
| 26 | 1671.793 | 3 | 5012.428 | -0.0725 | -14.5 | DAWWRFRKKRKRAKWCCCCCFEAFTRDDFRWRRRR |
| 27 | 1658.039 | 3 | 4971.139 | -0.0446 | -9 | DVGHRVRPWKKAKWKYMTSPLWWWMWWMMMCCCCMD |
| 28 | 1620.527 | 3 | 4858.519 | 0.039 | 8 | YLDKWMECRRRRRQFKKKRACQRRRSPKCELYAGRN |
| 29 | 1612.878 | 3 | 4835.675 | -0.0631 | -13 | DAHTFLLWWSVVSWRRRFKRLAKRQWNHQKKRLLKMM |
| 30 | 1609.262 | 3 | 4824.832 | -0.0686 | -14.2 | DGSNHYCEWWFYQTWWWWWMPQTYYECKYMMDDD |
| 31 | 1608.382 | 3 | 4822.054 | 0.0707 | 14.6 | CLGKKQLRLWYRRLRRRRGVKKLLHKKLLLLKKKKGAPS |
| 32 | 1608.056 | 3 | 4821.159 | -0.0134 | -2.8 | NYMPRGNDWWWHPDWRRRRRLWWWEGFVMHDGCMV |
| 33 | 1606.814 | 3 | 4817.355 | 0.0636 | 13.2 | KLRARRKRKLGVKKYQQRRRMMWWWYCCCFECMM |
| 34 | 1584.689 | 3 | 4751.109 | -0.0629 | -13.2 | DMNQEDDDMTWGVDRKKRRRRGALAVRFPGESCCEGYMC |
| 35 | 1565.407 | 3 | 4693.159 | 0.0398 | 8.5 | MLQWWSVWYYWLYEWCCRRVWWWYFQRAWWK |
| 36 | 1558.05 | 3 | 4671.197 | -0.0676 | -14.5 | DNYMMMEDASAAWRMYERRRNWWFKKRVLHKLMTGT |
| 37 | 1555.122 | 3 | 4662.318 | 0.0267 | 5.7 | LLSTVYRRRLAKRVCYCYWRAWWGEWYCYFLLTPA |
| 38 | 1549.513 | 3 | 4645.51 | 0.0088 | 1.9 | DAHTKQWAKKKKVLLLPLLLVCMDDHPKGVKTEKKQCVM |
| 39 | 1536.471 | 3 | 4606.195 | 0.1966 | 42.6 | KKLRVVVVVVVLSSTGDMECCCCCCAWWWKKALLYNE |
| 40 | 1520.909 | 3 | 4559.754 | -0.0485 | -10.6 | MRHYYNCSWMWWWHWWGAWAWYCEQYYNCDF |
| 41 | 1499.344 | 3 | 4495.044 | -0.0345 | -7.7 | FAKYKCRRALTHEGRDCSCDNWCEVDMLRLARYVC |
| 42 | 1498.858 | 3 | 4493.485 | 0.0669 | 14.9 | FKKKKWRRKKKWWWKWRHETGWKKQPFKHVAAC |
| 43 | 1489.053 | 3 | 4464.071 | 0.0654 | 14.6 | NYMRDRTRQKMWMHRMRKLLLLCRMMYCTPGDH |
| 44 | 1478.171 | 3 | 4431.429 | 0.0635 | 14.3 | DHMGLLVVLMRAQWWFHLRHKKRRVPKVGRFLCVF |
| 45 | 1471.223 | 3 | 4410.663 | -0.016 | -3.6 | DPPDKKKLRRPKCCCCHCCCSWWEGCCCECMM |
| 46 | 1463.511 | 3 | 4387.569 | -0.0563 | -12.8 | DCECDCCCEWWWCCCCFCMWRRRRLVMQM |
| 47 | 1458.164 | 3 | 4371.529 | -0.0584 | -13.3 | DCMMGWVYSCVYCCCCCCCCCCCRQRKKGQK |
| 48 | 1455.048 | 3 | 4362.239 | -0.1182 | -27.1 | QCCCDNWPCADSAMECCCCCAEGSASCCGDEDFCD |
| 49 | 1451.587 | 3 | 4351.68 | 0.0582 | 13.4 | KLFGRRLLRLPSLLDMLLLLLRRVFKKKRKLLETC |
| 50 | 1447.076 | 3 | 4338.149 | 0.0569 | 13.1 | DRTDAYCDACCYYMASVCRRKRKPRRRRRRRK |
| 51 | 1443.421 | 3 | 4327.274 | -0.0326 | -7.5 | DATHKKKVRRRYMPEWTTMHMGQKKKKKKHLCMV |
| 52 | 1430.936 | 3 | 4289.74 | 0.0453 | 10.5 | DAKKDACCCDDWWWYQLTYERTCCCCQQPAKK |
| 53 | 1421.977 | 3 | 4262.963 | -0.0534 | -12.5 | DAWMGAMACHEGWWWWWWWMRKRRRERRPR |
| 54 | 1421.973 | 3 | 4262.935 | -0.038 | -8.9 | DAWWWKNRRMMMMDCCCCGNPGVSGVKRRRVKK |
| 55 | 1421.982 | 3 | 4262.87 | 0.0548 | 12.8 | KLLVLHLRLLLGEWWWWWCCCCCCRMMCDF |
| 56 | 1421.638 | 3 | 4261.831 | 0.0608 | 14.2 | KWERRAWAARREHCMEEDWFHNENPYYCCLF |
| 57 | 1421.31 | 3 | 4260.85 | 0.0596 | 14 | VPLLLLRSNRMKWMECSMMWWERHMECCCRC |
| 58 | 1407.192 | 3 | 4218.538 | 0.0157 | 3.7 | DAHTWWWHGDMGPDFQNYMWEGWWYCCCYEN |
| 59 | 1403.28 | 3 | 4206.762 | 0.0556 | 13.2 | FNYMCMMDMWDACCRRMRELHKPDMKKLYNE |
| 60 | 1394.918 | 3 | 4181.669 | 0.0625 | 14.9 | DAHTRHGAGAKKWWWWCWWWFFCCCCMCVM |
| 61 | 1382.109 | 3 | 4143.359 | -0.0549 | -13.3 | DDAYCMCEYWMDSRRFAQWCCCCCCMGETC |
| 62 | 1377.673 | 3 | 4130.052 | -0.0555 | -13.4 | DMDMRYMMQQYRKRRRRRMKKKLRRQSGMM |
| 63 | 1370.152 | 3 | 4107.384 | 0.0509 | 12.4 | DAWMWTTTCCDEQCCECPWEGCCMTAGEEWW |
| 64 | 1368.679 | 3 | 4102.957 | 0.0597 | 14.5 | LLKKLREPRHKWWWCCFCDRHWWWKCVM |
| 65 | 1357.621 | 3 | 4069.894 | -0.0544 | -13.4 | DADACYMEYRRRRRRRFRRRKQYYCCFC |
| 66 | 1351.401 | 3 | 4051.126 | 0.0546 | 13.5 | VKPVEDYDPGRRRRRRKFKRRRKLDDSDYEC |
| 67 | 1350.388 | 3 | 4048.152 | -0.0118 | -2.9 | DAHTWLPKYKVRLRRRRRRRALKGEYMDCMV |
| 68 | 1337.884 | 3 | 4010.66 | -0.0286 | -7.1 | DCEDMMMDYNYNKNRFLGPEEQYKAAEVLVCM |
| 69 | 1321.992 | 3 | 3962.996 | -0.0415 | -10.5 | FHRFLRRWWWSVWWWMDPKKLLLTPAFCD |
| 70 | 1321.987 | 3 | 3962.989 | -0.0485 | -12.2 | DAHTYMEKKKVLRLLLRRRPEELAQCCQCVM |
| 71 | 1321.981 | 3 | 3962.964 | -0.0411 | -10.4 | LLTTLLRKLWPRRHKLMLMMMVHQCFAMDDD |
| 72 | 1321.982 | 3 | 3962.953 | -0.0302 | -7.6 | DAWQRKLLLLLVGGVQWWYCCCMPPPLVVMKD |
| 73 | 1321.987 | 3 | 3962.939 | -0.0009 | -0.2 | WLAPLKREERRDYMCMDMYKFVYFMRKLR |
| 74 | 1321.985 | 3 | 3962.888 | 0.0459 | 11.6 | LLGVKGVPVRRRRRRRKPSMECMMMWCCMM |
| 75 | 1321.979 | 3 | 3962.884 | 0.0312 | 7.9 | KLSKKKKKRPVKQAMMMWWWQCNECCNMGK |
| 76 | 1321.985 | 3 | 3962.879 | 0.053 | 13.4 | DAWKCGFWWCCCCWWMMMRKKLLLVLLLL |
| 77 | 1321.667 | 3 | 3961.953 | 0.0256 | 6.5 | LKKKKKKKLPTWWWWWWWYRMCCQCVM |
| 78 | 1321.317 | 3 | 3960.869 | 0.0593 | 15 | LAGKKKKKKFKKLWWWGECYDRAYCCCGMC |
| 79 | 1976.047 | 2 | 3950.04 | 0.0399 | 10.1 | RLLVARYKKRRRRRRRWGEMLCSWYCYC |
| 80 | 1317.01 | 3 | 3948.064 | -0.0568 | -14.4 | DACQTCLVSWWCRRRRRRKGVRLLRVVYNE |
| 81 | 1314.375 | 3 | 3940.051 | 0.0527 | 13.4 | DAHTWDDWRKKVKSVVVLKWEGWRGLRALCMV |
| 82 | 1964.155 | 2 | 3926.347 | -0.0507 | -12.9 | DCMGMDHLWWSVWWFMCCDRCCMMEETC |
| 83 | 1960.57 | 2 | 3919.078 | 0.0463 | 11.8 | DKKKKVKLRHKLLLRRLLLLMSDDYCECCDG |
| 84 | 1305.153 | 3 | 3912.378 | 0.0578 | 14.8 | LLLLPHKLLKLLLLLLLRALWQCFQLLLVGAMM |
| 85 | 1940.644 | 2 | 3879.331 | -0.0577 | -14.9 | DDNQHLGWWWYYCSSAACMYCMMEDDCYM |
| 86 | 1291.213 | 3 | 3870.623 | -0.006 | -1.6 | DHMGCCCCCHDDWQRQQRRVVRVLYESNF |
| 87 | 1278.868 | 3 | 3833.591 | -0.0075 | -1.9 | DCCECCCCNDGEWHRRRELWLYRSGKKGS |
| 88 | 1274.554 | 3 | 3820.654 | -0.013 | -3.4 | DAHTRAARLLPAVSNCCCHNWYYRYSSSCVM |
| 89 | 1272.111 | 3 | 3813.335 | -0.0239 | -6.3 | DPPDYFKFMMYCCCCCCMYMYVFQYNE |
| 90 | 1892.693 | 2 | 3783.422 | -0.0508 | -13.4 | DMMTCHGGGRFLKKKKCCCCCGAYCMCGGDS |
| 91 | 1887.311 | 2 | 3772.556 | 0.0512 | 13.6 | DAHTKAAQYMMCHWWCGECCQHLKKVEYN |
| 92 | 1881.899 | 2 | 3761.756 | 0.0272 | 7.2 | WLHLLWLWVSWWWWMRYLTTSGFQCTMG |
| 93 | 1880.527 | 2 | 3759.06 | -0.0213 | -5.7 | DAHTHPVSWPRRRRAMKKRRTKKKLVDEYN |
| 94 | 1875.575 | 2 | 3749.084 | 0.052 | 13.8 | LKPPKRRRVAFRRLKRYYWDKMHKVYEN |
| 95 | 1246.446 | 3 | 3736.276 | 0.0401 | 10.7 | VLKAPKCMDRDYCCCCCCCCDYMGGCVM |
| 96 | 1868.94 | 2 | 3735.823 | 0.0424 | 11.3 | WLCYYRRRFKKKRKLRGETGENCCCGSPA |
| 97 | 1245.405 | 3 | 3733.235 | -0.0408 | -10.9 | DAYCNCCCCCCCCCDGAFEERGAALLGDSY |
| 98 | 1862.022 | 2 | 3722.068 | -0.0391 | -10.5 | DATHYYCCQCCCCCCCCGGPQCCCYEN |
| 99 | 1226.133 | 3 | 3675.4 | -0.023 | -6.3 | DFTMDDCCCHSRRHKDGEKLDMEHEECY |
| 100 | 1225.54 | 3 | 3673.606 | -0.0088 | -2.4 | DPDLGPEDDDDNDSVWKCRRRRRPGPEMMQ |
| 101 | 1834.176 | 2 | 3666.209 | 0.1278 | 34.8 | QVSSVRFKQRAFKKKKKLLLFKQRATPPAALA |
| 102 | 1221.99 | 3 | 3663.002 | -0.0537 | -14.6 | DMGDYDLCCMGECCDDCMCDNEEQCDDC |
| 103 | 1221.986 | 3 | 3662.97 | -0.0334 | -9.1 | DACDDHMCCCCCCHNWCCPCWCCCC |
| 104 | 1221.987 | 3 | 3662.967 | -0.0277 | -7.6 | DCCEYWYMYRLRRRRRKKKKKRRQK |
| 105 | 1221.996 | 3 | 3662.914 | 0.0529 | 14.4 | LLLLYFEEWWWWTRWRFMRKYSRRR |
| 106 | 1221.989 | 3 | 3662.899 | 0.0451 | 12.3 | LRFKKKKKKAWKRWWWRWYTYDCGSGD |
| 107 | 1221.989 | 3 | 3662.897 | 0.0479 | 13.1 | DVGTGGPKWWWWWWWKRNRRARYRVKF |
| 108 | 1221.988 | 3 | 3662.891 | 0.0496 | 13.5 | DWRRRRHWWWWWWRHQYRRRRTR |
| 109 | 1831.879 | 2 | 3661.796 | -0.0538 | -14.7 | DVPERNLTRMYWMRRRELRFLTRNSGMM |
| 110 | 1827.116 | 2 | 3652.199 | 0.0182 | 5 | DVHGRRRKVLLLRLLGVRVGRRPAWRKMMK |
| 111 | 1213.565 | 3 | 3637.726 | -0.0523 | -14.4 | DAAVVLPPCRRKQWYWESEEETSGGKKYDPG |
| 112 | 1812.254 | 2 | 3622.493 | -0.0003 | -0.1 | DVVPLGERKKKLVYTCMTCGCCCCHMDDD |
| 113 | 1807.924 | 2 | 3613.804 | 0.0303 | 8.4 | LAGVPPKSVWWYFEFKLLLLVPWCMCDML |
| 114 | 1204.497 | 3 | 3610.517 | -0.0476 | -13.2 | DNYYCCSMDDSWWRRPRHRVVRESSMM |
| 115 | 1805.717 | 2 | 3609.467 | -0.0471 | -13 | DCGCYCMFMMGYDRDGYVKKRRKQYCM |
| 116 | 1203.538 | 3 | 3607.54 | 0.0528 | 14.6 | DWWVDKCPDNWWWWWWGECEERAAVR |
| 117 | 1200.57 | 3 | 3598.655 | 0.0329 | 9.1 | KLPVPLGELHQYCCQSWYRRFYYNYSGD |
| 118 | 1199.013 | 3 | 3593.971 | 0.0471 | 13.1 | LLLRMYMFDCRRRRRKKYKKKKVCVM |
| 119 | 1790.156 | 2 | 3578.245 | 0.052 | 14.5 | LLLVVKKLLKKKRRKRKGLVRVWSWWFD |
| 120 | 1192.857 | 3 | 3575.598 | -0.0486 | -13.6 | DATHWHTWWWSDGSMDWWRRRRRYNE |
| 121 | 1776.156 | 2 | 3550.343 | -0.0454 | -12.8 | DSSYMDPTVSWWWWWGEMYLYYCMCT |
| 122 | 1181.647 | 3 | 3541.971 | -0.0525 | -14.8 | DWCEEGCCQCCCCCCMWCYCCTDF |
| 123 | 1765.918 | 2 | 3529.845 | -0.0239 | -6.8 | DAEDWSCTPKSRRWARRRRRRLLPVSYG |
| 124 | 1765.112 | 2 | 3528.218 | -0.0092 | -2.6 | DDGAAMPAWWWWWWCCCCMYYGGMGMA |
| 125 | 1761.705 | 2 | 3521.438 | -0.0425 | -12.1 | DENNMEDDRSKRRDGACEECHGPGGQLEGFG |
| 126 | 1757.36 | 2 | 3512.663 | 0.0425 | 12.1 | WLFDWWWCCCRWYKLKKKKLTMGMA |
| 127 | 1752.357 | 2 | 3502.724 | -0.0247 | -7.1 | DVQWWWWYRRRRRRRRRGDHDCVM |
| 128 | 1751.936 | 2 | 3501.908 | -0.0509 | -14.5 | DMEPPGCCCDEDMDDACCCCCMMMDDD |
| 129 | 1749.534 | 2 | 3497.081 | -0.0283 | -8.1 | DCDNCNCCCYNPDWWWWCCMGGMGMA |
| 130 | 1747.672 | 2 | 3493.294 | 0.0363 | 10.4 | DDSCCMMWWWWWWGEWWGEFLHGND |
| 131 | 1742 | 2 | 3481.965 | 0.0193 | 5.5 | DHMGHLQRRRVKKVKKQARHGVRKKEYN |
| 132 | 1733.503 | 2 | 3464.972 | 0.0193 | 5.6 | DMHDCMMTCCMAYSMEDMMMMMCGFD |
| 133 | 1731.2 | 2 | 3460.391 | -0.0065 | -1.9 | DVGTHWWKNHPLCCMMMMGMVCELWYG |
| 134 | 1153.386 | 3 | 3457.112 | 0.0227 | 6.6 | DNKRKKKPGPRRFRKFLRRRRLLPLPE |
| 135 | 1724.934 | 2 | 3447.878 | -0.0245 | -7.1 | DYAYTPPRLLLLLPLLLYTRRVYYGFAMG |
| 136 | 1149.566 | 3 | 3445.631 | 0.0452 | 13.1 | RLYRAWWWWMRRERWWWWWPDH |
| 137 | 1701.416 | 2 | 3400.851 | -0.0338 | -9.9 | DACCCCPACCCCCCCCMMMMGGCVM |
| 138 | 1696.877 | 2 | 3391.693 | 0.0453 | 13.4 | DAVDAKLWFLRYCFEWRRWKKLCVM |
| 139 | 1127.159 | 3 | 3378.412 | 0.0443 | 13.1 | DAWWWWWWWWWWYWWFWREC |
| 140 | 1685.656 | 2 | 3369.345 | -0.0473 | -14 | RLCMQCWWWWWWWWWEYGGCFD |
| 141 | 1681.791 | 2 | 3361.562 | 0.0051 | 1.5 | LLLLKRRRADYCMCCEPPRMPYCCK |
| 142 | 1680.622 | 2 | 3359.181 | 0.0478 | 14.2 | DEDGCCMDWWWWWWWGYEMEGWGM |
| 143 | 1120.534 | 3 | 3358.628 | -0.0486 | -14.5 | WLPLLLKKQWWPPEHYYQLPACWMC |
| 144 | 1666.402 | 2 | 3330.753 | 0.0356 | 10.7 | LHEEGWYLKKWRRRRFRKLCEDRN |
| 145 | 1109.983 | 3 | 3326.879 | 0.048 | 14.4 | LLRWWEVGWALQRRRRRRGVQYMKK |
| 146 | 1658.76 | 2 | 3315.552 | -0.046 | -13.9 | DVMDWWMECMPEKKRRRVLALLCMV |
| 147 | 1654.184 | 2 | 3306.241 | 0.1133 | 34.3 | VLLLLRRRAYKKKKRRSVVKKKKKKR |
| 148 | 1633.13 | 2 | 3264.294 | -0.0478 | -14.6 | DNGCMMDGVSDMDQRMVYWQRMMVVF |
| 149 | 1632.182 | 2 | 3262.345 | 0.0037 | 1.1 | YMNEEDERFWWWRRWQYMGGGGEY |
| 150 | 1629.558 | 2 | 3257.017 | 0.0838 | 25.7 | PLLLLLLLLRLLYYDVKRRRRFKTW |
| 151 | 1085.889 | 3 | 3254.6 | 0.0466 | 14.3 | GGYMWWWYPHDWKRRRKRRLGADY |
| 152 | 1627.984 | 2 | 3253.907 | 0.0467 | 14.3 | DTSKVSKKKKAAGGKLLLVRRRRPGVCVM |
| 153 | 1622.468 | 2 | 3242.963 | -0.0408 | -12.6 | DDNQMEDDFHVCGHDCCCMCMCDNS |
| 154 | 1621.424 | 2 | 3240.877 | -0.0447 | -13.8 | DLMPCCCMMDCCCENCACGDDDDDM |
| 155 | 1077.386 | 3 | 3229.166 | -0.0304 | -9.4 | DAWCMMECEGMMCCSEWQSVWHKK |
| 156 | 1610.958 | 2 | 3219.947 | -0.0444 | -13.8 | DEDECCERHNCCECCEMACEEYC |
| 157 | 1609.987 | 2 | 3217.998 | -0.0392 | -12.2 | CLAAQMCCCCCMMMFCCEHLMQM |
| 158 | 1608.971 | 2 | 3215.887 | 0.0408 | 12.7 | MRKALKKWCRFRRRTTGKWRLLRL |
| 159 | 1608.41 | 2 | 3214.76 | 0.0442 | 13.7 | DAYYCGVKHLAWWRRRKWRLLLGVL |
| 160 | 1608.359 | 2 | 3214.749 | -0.0468 | -14.6 | DLLVPMYMAWWWWWHKKKKKKVVL |
| 161 | 1608.375 | 2 | 3214.689 | 0.0463 | 14.4 | DVPERHDKKLLTGLLLTAPRLRHDCMM |
| 162 | 1608.359 | 2 | 3214.658 | 0.0456 | 14.2 | KYRRKWWWWWWWHCRRVRAAR |
| 163 | 1608.039 | 2 | 3214.097 | -0.0342 | -10.6 | FNYCEDWWSVWEGCCCFCEDYNE |
| 164 | 1607.867 | 2 | 3213.673 | 0.0458 | 14.2 | KKKKKKKLRRKRMECKYFMCMNM |
| 165 | 1606.308 | 2 | 3210.574 | 0.0273 | 8.5 | DPEVSVWWCCFNKKKKLPLELLMQM |
| 166 | 1605.145 | 2 | 3208.315 | -0.0395 | -12.3 | FYNDSYKQAKLDKMHEQYYCGGCCQ |
| 167 | 1603.696 | 2 | 3205.368 | 0.0093 | 2.9 | RLGPKLLPSWWWWWWYCCCMGDM |
| 168 | 1600.66 | 2 | 3199.327 | -0.0209 | -6.5 | MYNWWYCCCCVSYQYRELWTKK |
| 169 | 1598.781 | 2 | 3195.517 | 0.0294 | 9.2 | RLEWWCCWCCEGKSVCRKKKLGPL |
| 170 | 1061.676 | 3 | 3182.053 | -0.0457 | -14.4 | MRMQLGKWCMMCCCCCCCHMDK |
| 171 | 1586.195 | 2 | 3170.412 | -0.0364 | -11.5 | DACCCHHWWCCRHLVRAYRRGVR |
| 172 | 1576.317 | 2 | 3150.665 | -0.0465 | -14.8 | DQECCHFFLLLLLLVVLPLELLYGPH |
| 173 | 1048.54 | 3 | 3142.558 | 0.0393 | 12.5 | DPPDWWWWWWRRRRPSKKLYNE |
| 174 | 1569.52 | 2 | 3137.072 | -0.0456 | -14.5 | WYCCACYDMMMMESWWWRYDGS |
| 175 | 1043.968 | 3 | 3128.922 | -0.0394 | -12.6 | DCMDNMEGTMGMREEMGDCEDDCMM |
| 176 | 1564.681 | 2 | 3127.39 | -0.0433 | -13.8 | DATHYFNWWWYESVWYKRKGMGMA |
| 177 | 1561.229 | 2 | 3120.399 | 0.0454 | 14.5 | DMFWWWWWYEGERRKPSRLMPM |
| 178 | 1554.924 | 2 | 3107.809 | 0.0252 | 8.1 | DLALPAPHDRRRRKLLLRRVVPAMKD |
| 179 | 1552.2 | 2 | 3102.432 | -0.0462 | -14.9 | DAWWWWWFSGCCEDRCRKKKKK |
| 180 | 1034.646 | 3 | 3100.891 | 0.0247 | 8 | DAPADPKWQLRRRRRKKKKKKRQK |
| 181 | 1548.967 | 2 | 3095.875 | 0.0442 | 14.3 | AKLYRRLVPPGPKLLYLRRRKLMVSG |
| 182 | 1548.502 | 2 | 3094.983 | 0.0065 | 2.1 | DPPDYYYCCCECGEEGCYYNMMQ |
| 183 | 1546.034 | 2 | 3090.082 | -0.0283 | -9.2 | DMGHWYCCCCCCCMKLRPVMQM |
| 184 | 1027.286 | 3 | 3078.877 | -0.0423 | -13.7 | DCSSMDPSDDMCRMMMDEEGMMMC |
| 185 | 1539.395 | 2 | 3076.818 | -0.042 | -13.7 | DEEDDEDDCCYCCMCMGDNDYCM |
| 186 | 1535.615 | 2 | 3069.226 | -0.0113 | -3.7 | DWAEGYCCCCCMRRDSHSMKKKL |
| 187 | 1527.926 | 2 | 3053.805 | 0.0332 | 10.9 | DTRRLLRRRGMVLLLLLLVVVQGMTT |
| 188 | 1015.939 | 3 | 3044.831 | -0.037 | -12.1 | DHESVWMKKLLKVFKLLLLKHRKR |
| 189 | 1522.815 | 2 | 3043.579 | 0.0357 | 11.7 | CLTWEGWWGAFERPPRKKKKKCPV |
| 190 | 1521.964 | 2 | 3041.947 | -0.0334 | -11 | DPCCCCCCCCDLLLRCCCMYM |
| 191 | 1521.965 | 2 | 3041.871 | 0.0453 | 14.9 | KLAYCFKKKKLLGEVGFKKKKKLFL |
| 192 | 1518.206 | 2 | 3034.372 | 0.0245 | 8.1 | DLRAWWWWLWGEEGYKMVWSGYM |
| 193 | 1516.237 | 2 | 3030.424 | 0.0359 | 11.8 | FKGYWYGYVWWALMYKEFTWYK |
| 194 | 1516.226 | 2 | 3030.394 | 0.0442 | 14.6 | DVHHLDAHLLVHSSCTGCGMYFTKFV |
| 195 | 1513.695 | 2 | 3025.338 | 0.0379 | 12.5 | DEQCCCWMYYMEPNERKKKVKK |
| 196 | 1009.143 | 3 | 3024.446 | -0.0374 | -12.4 | DNYYCMMRRRRRQKVKLYYMY |
| 197 | 1507.916 | 2 | 3013.851 | -0.0332 | -11 | DAHTWCCCCPCCCCCCHEMQM |
| 198 | 1502.266 | 2 | 3002.477 | 0.0403 | 13.4 | DVPEWWWWRRRRRQPSRQHGND |
| 199 | 1001.384 | 3 | 3001.099 | 0.0321 | 10.7 | DATHMHGDWWFMWNEMEMHEGYN |
